# Relational-responsive music sharing and third-party charitable allocation: a 7-day randomized field experiment on social media

**DOI:** 10.3389/fpsyg.2026.1932126

**Published:** 2026-08-28

**Authors:** Tao Liu, Xin Wang

**Affiliations:** 1 College of Arts, Xiamen University, Xiamen, Fujian, China; 2 School of Journalism and Communication, Chongqing University, Chongqing, China

**Keywords:** charitable allocation, music sharing, perceived social presence, randomized field experiment, social grooming, social identity

## Abstract

**Background:**

Music listening and collective musical activity are linked to prosocial outcomes, yet everyday social-media music sharing is little studied. Using the social grooming model, we tested the overall effect of a multicomponent relational-responsive music-sharing bundle on hypothetical charitable allocation; the design did not isolate music or any single component.

**Methods:**

In a 7-day, three-arm randomized field experiment on WeChat Moments (583 randomized; mITT *N* = 568), the focal contrast compared relational-responsive music sharing with informational posting under the same daily posting target (*n* = 377); a third arm used minimal music. The primary outcome was tokens allocated to two charities from a fixed 10-token endowment. Perceived social presence and social identity were measured concurrently at endpoint; perceived social support was an exploratory moderator.

**Results:**

The bundle increased allocation over informational posting [*M* = 4.08 vs. 3.28; *b* =0.758, 95% CI (0.377, 1.138), *d* = 0.425]; informational posting did not differ from minimal use. The serial presence → identity association was positive [0.053 (0.021, 0.099)]; presence-only and identity-only associations included zero. Unpaid helping did not differ [OR = 1.20 (0.79, 1.81)]. The bundle raised total allocation and shifted composition toward the music-education beneficiary.

**Conclusions:**

Assignment to the bundle causally raised hypothetical charitable allocation, but the design cannot show music was necessary or uniquely effective, concurrent measurement cannot establish mediator ordering, and the beneficiary shift is a descriptive composition change, not generalized prosociality.

## 1 Introduction

Music circulates widely across contemporary social media, where songs and playlists move through the feeds of friends and acquaintances and invite recognition, comment, and response. Research on online music sharing shows that these acts are shaped not only by ease of use but also by enjoyment, self-expression, social presence, and social identity (Lee et al., 2011; Liu and Luo, 2023). Qualitative work likewise shows that people share music selectively to express identity, affirm affinity, sustain existing ties, and open later conversation (Hagen and Lüders, 2017). Music sharing, therefore, carries a communicative logic that cannot be reduced to content distribution: it places musical taste within an interpersonal setting and gives it relational meaning.

Because it is expressive, music sharing can operate in two registers, and the difficulty of telling them apart motivates the present study. In one register, a shared song becomes a relational act, communicating taste, recognizing similarity, and sustaining connection; shared musical activity within friendships and romantic relationships is correspondingly associated with coordination, positive communication, and relationship commitment (Harwood and Wallace, 2022). In the other, sharing remains self-presentation, taste display, or ambient posting that leaves little relational trace. Social media platforms can accommodate both registers through interactive, low-friction sharing tools (Lee et al., 2011), while the outward form of a post seldom reveals which relational logic is operating. The distinction matters because public-interest communicators routinely use music to evoke empathy and mobilize action, and prosocial lyrics can increase charitable donation (Hong et al., 2023), yet the conditions under which shared music moves audiences to act remain unspecified. Existing research does not yet clearly distinguish relationally consequential music sharing from otherwise similar posting that leaves little relational trace.

Scholarship has extended the social grooming model across a widening range of media, from text to static photographs and dynamic visual formats, and has shown that grooming can generate social capital, perceived support, and wellbeing for the groomer (Lin, 2019; Lin and Hsieh, 2021; Liu and Yeo, 2024; Wang and Wang, 2025). What remains unclear is whether these relational returns extend outward to behavior that benefits people outside the exchange. The model reconceives routine online acts, including posting, disclosing, liking, commenting, and responding, as digital descendants of the physical grooming that sustains primate bonds: low-cost investments of attention that initiate, maintain, and strengthen interpersonal ties (Dunbar, 1996; Tufekci, 2008). Their relational force lies less in informational payload than in the attention they signal, since a like or a comment marks another person as worthy of notice (Donath, 2007; Wang and Wang, 2025). Music sharing has received comparatively little attention within this expansion, even though it possesses several qualities on which grooming can build. Musical taste communicates values and perceived similarity (Boer et al., 2011); shared musical activity supports coordination, positive communication, and relationship commitment (Harwood and Wallace, 2022); and theoretical work describes music as a shared experience of an embodied present (McGuiness and Overy, 2011). When these qualities are combined with personal framing and responsive interaction, a shared song can carry self-disclosure, directed attention, and personally meaningful or shared memories into an ongoing relationship. The present study, therefore, treats relational music sharing as a form of grooming and names it the relational-responsive music-sharing intervention bundle: the intentional and relational use of music sharing, music-related self-disclosure, and responsive interaction to maintain ties, signal care, evoke shared memories, and build social connection. The qualification carries weight because music sharing takes on this character only when it is relationally framed, intentionally addressed, and embedded in responsive interaction.

Read through the lens of grooming, three gaps in the literature come into view, and together they define the question the study poses. The first concerns the medium of grooming. Studies in this area has moved from establishing that ordinary online acts perform relationship maintenance toward broadening the media forms through which grooming operates and specifying the conditions of its effects (Lin, 2019; Wang and Wang, 2025). Its documented outcomes, however, have stayed self-directed, encompassing the groomer's own social capital, support, and well-being (Lin and Hsieh, 2021; Liu and Yeo, 2024; Wang and Wang, 2025). Music, among the widely shared materials online, has not been examined as a vehicle for grooming, and the question of whether grooming's benefits reach outward to behavior that serves other people has not been asked.

A second gap concerns the direction of explanation in research on music sharing, and it follows naturally from the first. That literature has progressed from music piracy and its industry effects toward the social psychology of legal sharing, and it has established that social presence and social identity motivate the intention to share, a pattern that holds across cultures (Lee et al., 2011; Liu and Luo, 2023). In this study, presence and identity are treated as reasons that people share; what an act of sharing then produces has been left untested. If presence and identity prompt sharing, a sustained period of relational sharing may in turn generate them, and this reciprocal possibility has yet to be considered.

A third gap concerns the setting in which music acquires prosocial force. Joint music-making can increase cooperation and helping (Kirschner and Tomasello, 2010), while group music participation has been associated with prosocial behavior through positive social connections and peer support (Yang et al., 2025). Across these strands, music's social consequences are closely tied to relational connection. Yet the evidence comes largely from physically co-present, performance-, or listening-based settings. Whether mediated music sharing, the everyday form that musical sociality often takes online, carries comparable prosocial consequences remains unclear.

These three gaps converge on a single question: can a relational-responsive interaction bundle conducted through music on social media promote prosocial behavior, are the observed endpoint associations consistent with the proposed roles of perceived social presence and social identity, and does any increase in giving extend across beneficiaries or concentrate within the music-related domain? The study extends research on music and prosocial behavior to a naturalistic social-media intervention, and it tests the bundle as a whole; it does not test the unique effect of music or of any single intervention component.

Two limits on the scope of this question should be stated before the framework is developed. First, the experiment contrasts a relational-responsive music-sharing bundle with informational music posting, and it contains no relational non-musical arm; it therefore asks what relational-responsive sharing conducted through music produces, and it cannot ask whether music adds anything to relational communication carried by other content. Second, the outcome is a fixed-budget allocation between the participant and two named charitable beneficiaries; it therefore indexes stated allocation toward third parties and the distribution of that allocation across beneficiaries, and not prosocial disposition in general. Both limits guide the operationalization, the analyses, and the interpretation.

The framework proposes a theoretically ordered relational pathway and one exploratory boundary condition. Perceived social presence refers to the sense of being together with another person and of mutual attention in mediated interaction (Biocca et al., 2003; Harms and Biocca, 2004). Relational-responsive sharing may heighten this perception because attention given and received renders each party salient as an attending person, while the shared affect and perceived coordination associated with musical experience give that attention expressive and relational content (McGuiness and Overy, 2011; Harwood and Wallace, 2022). Social identity refers here to belonging, closeness, and positive feelings toward the people encountered during the intervention week, drawing on Lee et al. (2011). The model places perceived social presence before social identity and treats charitable allocation to third-party beneficiaries as the outcome. Perceived social support is examined as an exploratory boundary condition.

Self-categorization theory holds that identification develops when perceived similarities become salient and meaningful within the immediate context (Tajfel and Turner, 1979; Turner, 1985). In mediated interaction, social presence supplies awareness of others and mutual attention (Biocca et al., 2003; Harms and Biocca, 2004), giving musical similarity a relational setting in which it can matter. Online music-sharing research has linked interactivity and self-expression with social presence and has identified both social presence and social identity as predictors of sharing intention (Lee et al., 2011; Liu and Luo, 2023). Reversing the direction of inquiry, the present framework treats repeated presence-laden exchange as a condition through which identification with interaction partners can emerge. Once formed, identity can shape target-specific social valuation and allocation (Hackel et al., 2017).

Perceived social support, the perceived availability of support from significant others, family, and friends (Zimet et al., 1988), is examined as a relational condition under which the sequence may unfold, since whether a shared song is experienced as care or as noise plausibly depends on how supported its recipient already feels. The outcome, prosocial behavior, is voluntary behavior intended to benefit others and encompasses helping, sharing, caring, and giving (Carlo and Randall, 2002; Caprara et al., 2005). In sum, the framework proposes that a relational-responsive communication practice may influence prosocial behavior through two relational states and examines perceived social support as a possible boundary condition.

Four contributions follow. First, the study extends research on music and prosociality beyond listening, lyrics, synchrony, and co-present music-making by examining whether music shared in everyday social-media interaction can influence a prosocial outcome. Second, it examines a relational-responsive music-sharing bundle, understood through social grooming theory, as one form of mediated musical interaction, complementing grooming accounts built on text and images (Lin, 2019; Wang and Wang, 2025). Third, it tests a proposed pathway in which perceived social presence and social identity, previously examined as antecedents of music-sharing intention (Lee et al., 2011; Liu and Luo, 2023), are considered as relational states associated with the behavioral outcome after sharing; because both were measured concurrently, the study evaluates an ordered indirect association, and it does not demonstrate a temporal mechanism. Fourth, it separates the overall level of prosocial allocation from its composition across beneficiaries and treats perceived social support as an exploratory boundary condition.

## 2 Hypothesis development

### 2.1 Music and prosocial behavior

Music is a human universal, present in every known culture, and influential evolutionary accounts locate its significance in social bonding and cooperation (Cross, 2001; Huron, 2001; Mehr et al., 2019; Savage et al., 2021). Helping, volunteering, sharing, and giving are behavioral expressions of this social orientation, so the question of how music promotes prosocial action bears directly on its proposed social function. Research on this question can be organized by what, within musical experience, is expected to do the prosocial work: lyrical content, the emotions music induces, the synchrony it affords, and the identity it expresses. These mechanisms need not operate in isolation; a recent synthesis conceptualizes them as complementary processes of perceptual alignment that shift experience from a self-oriented toward a relational mode (Chen et al., 2026).

Prosocial behavior, voluntary action intended to benefit others, encompasses helping, sharing, caring, cooperating, and donating (Eisenberg and Miller, 1987; Carlo and Randall, 2002). Empathy is a central proximal driver because the capacity to perceive and share others' emotional states repeatedly predicts helping and cooperation (Batson et al., 2007; Roberts and Strayer, 1996; Eisenberg and Miller, 1987). Situational affect is a second driver: sadness can shift welfare-oriented judgments and increase willingness to devote time to helping others (Small and Lerner, 2008; Yang et al., 2017). Group membership is a third: people favor in-group targets in their allocations, and this bias is graded by how strongly they identify with the group (Hackel et al., 2017). Empathy, affect, and identification are therefore precisely the levers through which music can acquire prosocial force (Wu et al., 2025; Chen et al., 2026).

The clearest evidence concerns prosocial lyrics. Listening to prosocial rather than neutral lyrics can increase prosocial thoughts and affect and has been followed by greater helping (Greitemeyer, 2009a), monetary donation (Greitemeyer, 2009b; Hong et al., 2023), tipping (Jacob et al., 2010), and everyday helping (Greitemeyer, 2011a; Kennedy, 2013). The pattern is not uniform: Ruth and Schramm (2021) found effects on prosocial thoughts and empathic emotions but not charitable donation. Prosocial lyrics have also been linked to reduced aggression (Greitemeyer, 2011b), reduced prejudice and discrimination (Greitemeyer and Schwab, 2014), and greater generosity in intertemporal contexts (Hong et al., 2025). Their effects depend partly on how listeners attend to and recognize the music, including familiarity and empathic engagement (Ruth, 2019), while production features can further shape the emotional reception of prosocial lyrics (Ruth and Schramm, 2021).

Whether music independent of lyrics produces prosocial effects is less settled. Some evidence is affirmative: soothing and stimulating music increased helping relative to aversive music or silence (Fried and Berkowitz, 1979), and chill-inducing music enhanced altruism (Fukui and Toyoshima, 2014). Other evidence is null: uplifting instrumental music did not increase donations over silence (Ganser and Huda, 2010), and when lyrics were neutral, the presence of music did not affect prosocial decisions (Yu et al., 2019). Taken together, these findings suggest that exposure to music alone is insufficient and direct attention toward the affective qualities of the experience. Controlled work on mode and tempo supports this view: mode-related mood influenced helping decisions, whereas tempo-related arousal did not (Wu et al., 2025). The following sections, therefore, consider emotion alongside coordination and identity as routes through which music may acquire prosocial force.

Affect provides one route from music to prosocial decision-making. The two-dimensional model of affect distinguishes mood from arousal (Russell, 1980), and music can elicit states that differ along both dimensions (Palazzi et al., 2019). In Western tonal music, mode and tempo are especially influential, with major and fast music typically evoking happiness and minor and slow music evoking sadness (Krumhansl, 1997; Peretz et al., 1998). When these features are separated, mode-related mood, but not tempo-related arousal, changes willingness to help, with sad music producing greater helping than happy music or noise (Wu et al., 2025). This pattern converges with broader evidence that sadness can orient social judgment and helping intentions toward others (Small and Lerner, 2008; Yang et al., 2017).

Empathy provides the interpersonal bridge between musical emotion and prosocial behavior (Clarke et al., 2015; Miu and Vuoskoski, 2017). It is the process by which people perceive and resonate with others' emotional states (Preston and de Waal, 2002) and a well-established driver of prosocial action (Batson et al., 2007; Eisenberg and Miller, 1987; Roberts and Strayer, 1996). Musical emotion can emerge through processes such as emotional contagion and appraisal (Juslin and Västfjäll, 2008; Scherer and Zentner, 2001), while individual empathy shapes the relation between recognized and felt musical emotion (Egermann and McAdams, 2013). These processes make state empathy toward a target, distinct from trait empathy (Cuff et al., 2016), a plausible route from music-induced emotion to helping. Controlled evidence further shows that mode-related mood influences helping decisions, whereas Theory of Mind and memory strength do not account for the same effect (Wu et al., 2025). Evidence from longer-term musical experience is mixed: training and musical sophistication have been associated with cognitive empathy in some samples (Hua et al., 2025), but musical practice does not consistently distinguish empathy or prosociality (Morand-Grondin et al., 2026). Music-induced emotion also affects adjacent decision domains, including risk-taking and intertemporal choice (Schulreich et al., 2014; Israel et al., 2019), situating prosocial decision-making within a broader pattern of affect-sensitive judgment. Because musical pieces differ across multiple acoustic dimensions, controlled manipulations of mode and tempo remain especially informative for isolating affective effects (Wu et al., 2025).

A second mechanism is interpersonal coordination. Music induces temporal synchrony, and synchronized movement increases liking, cooperation, and compliance between people (Mogan et al., 2017; Wiltermuth and Heath, 2009). Coordinated musical activity is theorized to produce “self-other merging,” blurring the boundary between self and partner and thereby strengthening interpersonal bonds (Tarr et al., 2014). Group singing raises social closeness and collective cohesion, accompanied by physiological changes such as elevated pain thresholds (Weinstein et al., 2016), and interpersonal synchrony in musical contexts promotes bonding through coordinated activity and shared physiological response (Stupacher et al., 2022; Yu et al., 2025). Even when people are not moving, co-experiencing music is associated with perceptions of coordination and a shared experiential present (McGuiness and Overy, 2011; Harwood and Wallace, 2022). This coordination has behavioral consequences: synchrony has been linked to greater prosociality and cooperation (Mogan et al., 2017; Wiltermuth and Heath, 2009), and synchronized movement has enhanced memory for co-participants (Woolhouse et al., 2016). A complementary neuroendocrine account proposes that oxytocin and testosterone may help connect cooperative musical behavior with affiliation and bonding (Fukui and Toyoshima, 2023). The affective and synchrony mechanisms are not wholly separate: shared emotional experience and coordinated timing are argued to converge on the same relational outcome, interpersonal closeness (Chen et al., 2026).

A third mechanism runs through identity. Musical taste functions as a social cue: shared preferences communicate value similarity and increase social attraction (Boer et al., 2011), while shared musical activity is associated with coordination, positive communication, and relationship commitment (Harwood and Wallace, 2022). These processes can turn musical affinity into a basis for collective belonging. Once salient, social identity shapes target-specific valuation and allocation, with stronger identification increasing preference for relevant others (Hackel et al., 2017). Longitudinal work on red music further connects musical identity with awe and prosocial behavior within an indirect pathway to later wellbeing (He et al., 2025). Identity and empathy may also converge: interpersonal closeness is associated with stronger affect sharing (Lin et al., 2024), while empathy can generate social closeness that persists over time (Saulin et al., 2024). Together, these links help explain why identity-based and affective routes to prosociality can become intertwined (Chen et al., 2026).

The evidence reviewed above comes almost entirely from listening, lyrical exposure, synchrony, and co-present music-making; whether relationally shared music on social media produces comparable behavioral consequences has not been tested.

Across all three mechanisms, the evidence rests largely on music that is listened to, performed, or experienced in shared physical space. Lyric effects are established through listening to researcher-selected songs (Greitemeyer, 2009b); affective effects through controlled listening (Wu et al., 2025); synchrony and bonding through joint singing and coordinated movement (Weinstein et al., 2016; Tarr et al., 2014); and identity-related effects through shared taste and social attraction (Boer et al., 2011). Naturalistic research broadens the picture: group music participation is associated with prosocial behavior through positive social connections and peer support (Yang et al., 2025), and shared musical activity within close relationships is associated with commitment through coordination and positive communication (Harwood and Wallace, 2022). Online shared-listening experiments move one step further, showing that simulated social listening can heighten musical pleasure and that this pleasure is associated with more generous Ultimatum-game offers (Curzel et al., 2024). These findings show that mediated co-experience can alter how music is felt, but they do not yet address user-generated, asynchronous sharing in an existing social network. The unresolved question is therefore whether such sharing can alter a later third-party allocation and how a relational-responsive bundle compares with informational posting. The inconsistent evidence for pure-music effects even under controlled listening (Ganser and Huda, 2010; Yu et al., 2019) makes this distinction especially important.

What the literature has not examined is music sharing as a route to prosocial behavior. Online music sharing is an everyday form of musical sociality shaped by enjoyment, self-expression, interactivity, social presence, and social identity (Lee et al., 2011; Liu and Luo, 2023). Unlike listening, sharing places musical content inside a communicative act whose relational force depends on how the post is framed and whether others respond. This distinction matters because established mechanisms generally presuppose either a controlled stimulus, such as lyrics or mode, or bodily co-presence, such as synchrony, whereas sharing relocates music into asynchronous network interaction. The present study, therefore, tests whether assignment to a relational-responsive music-sharing bundle changes hypothetical third-party charitable allocation relative to informational music posting.

To bridge listening and sharing, we treat relational music sharing as a form of social grooming: the intentional, relationally oriented use of self-disclosure and responsive interaction to maintain and strengthen ties (Dunbar, 1996; Tufekci, 2008). Within the present intervention, music sharing takes this form when participants add personal, emotional, or relational framing and engage in substantive response. The shared song brings emotional content and signals of taste and value similarity into an exchange whose responsiveness can acquire relational meaning (Boer et al., 2011; Harwood and Wallace, 2022; Wu et al., 2025). The connection generated in that exchange may also extend beyond the immediate partners. Across three experiments, making relatedness salient increased volunteering interest, general prosocial intentions, and charitable donation; felt connectedness carried the increase in general prosocial intentions (Pavey et al., 2011). Repeated relationally framed sharing may therefore shift participants from self-focused toward other-regarding allocation even when the beneficiaries are not the interaction partners. This argument predicts the total number of tokens allocated away from the self; by itself, it does not determine how those tokens will be distributed between beneficiaries.

These two routes are not merely alternatives; they imply different scopes of prosociality. The motivational route treats connection as raising a general other-regarding state, leaving the distribution of giving across beneficiaries open (Pavey et al., 2011). The identity route predicts target differentiation: identification changes the value assigned to socially relevant others (Hackel et al., 2017) and can organize cooperation and prosocial behavior across local, national, global, teammate, and opponent boundaries (Grimalda et al., 2023; Bruner et al., 2014). On this account, the same increase in total giving may be distributed unevenly when one beneficiary is more closely aligned with the domain made salient by the interaction. Existing work on music and prosociality has rarely had to distinguish these possibilities because outcomes have typically been measured toward interaction partners or an undifferentiated other (Kirschner and Tomasello, 2010; Yang et al., 2025). Offering participants two beneficiaries that differ in their relation to the musical domain makes the distinction testable, and H9a and H9b state the competing predictions that follow.

Two lines of evidence motivate a direct hypothesis. First, studies have linked prosocial lyrics to helping and charitable allocation (Greitemeyer, 2009a,b; Hong et al., 2023). Second, group music participation has been associated with prosocial behavior through positive social connections and peer support (Yang et al., 2025). If relational music sharing carries emotional, expressive, and relational content toward particular others, it may likewise increase prosocial behavior. We hypothesize:

- *H1: Assignment to the relational-responsive music-sharing intervention bundle increases hypothetical third-party charitable allocation relative to informational music posting*.

### 2.2 Perceived social presence

The direct effect should operate through relational states that mediated sharing can create. The first is perceived social presence, the sense of being with another person and of mutual attentional awareness in interaction (Biocca et al., 2003; Harms and Biocca, 2004). Presence translates the attentional structure of grooming into a felt interpersonal state. In online music-sharing research, interactivity and self-expression have been linked to social presence, while social presence and social identity predict sharing intention across cultural settings (Lee et al., 2011; Liu and Luo, 2023). Relational-responsive sharing should therefore make the exchange feel less like ambient posting and more like being with an attending other: personal framing gives the post a relational address, while substantive response makes mutual attention visible. Music can deepen this experience because shared musical experience carries affective alignment and perceived coordination (McGuiness and Overy, 2011; Harwood and Wallace, 2022). As awareness of another person becomes more immediate, the exchange should become more other-oriented, providing a mediated route from relational sharing to prosocial behavior. We hypothesize:

- *H2: Assignment to the relational-responsive music-sharing intervention bundle is positively and indirectly associated with hypothetical third-party charitable allocation through perceived social presence*.

### 2.3 Social identity

The second relational state is social identity, the knowledge of belonging to a group together with its emotional and value significance (Tajfel and Turner, 1979). Music gives this process a concrete basis: shared preferences communicate value similarity and social attraction (Boer et al., 2011), while shared musical activity is associated with coordination, positive communication, and relationship commitment (Harwood and Wallace, 2022). Repeated exchanges of personal musical disclosure and responsive attention should therefore convert perceived similarity into a sense of “we” among interaction partners. Once salient, social identity reorganizes target-specific valuation and allocation (Hackel et al., 2017). The association of group music participation with prosocial behavior through positive social connections and peer support further illustrates how musical affiliation can acquire behavioral consequences (Yang et al., 2025). We hypothesize:

- *H3: Assignment to the relational-responsive music-sharing intervention bundle is positively and indirectly associated with hypothetical third-party charitable allocation through social identity*.

Perceived social presence and social identity are hypothesized to be ordered. Presence is an immediate perception of the interaction; identity reflects a more enduring sense of belonging. Prior studies have reported positive paths from social presence to social identity (Shen et al., 2010; Lee et al., 2011), providing a basis for the proposed ordering. In the present model, relational music sharing is expected to increase perceived presence, presence is expected to strengthen social identity, and identity is expected to increase prosocial behavior. Because both mediators are measured concurrently, this sequence remains a theoretical hypothesis; it is not a directly observed temporal process. We hypothesize:

- *H4: Assignment to the relational-responsive music-sharing intervention bundle is positively and indirectly associated with hypothetical third-party charitable allocation serially through perceived social presence and then social identity*.

### 2.4 Perceived social support

Perceived social support may shape how participants interpret relational overtures. Under an enrichment account, people who perceive stronger support may be more likely to read a shared song and a substantive response as signals of care and reciprocity. Under a compensation account, relational-responsive sharing may matter more to people with fewer existing relational resources because it supplies a form of connection that is otherwise less available. These alternatives imply different moderation patterns, and neither has been established for the present context.

The available music literature does not resolve this issue. Group music participation has been associated with prosocial behavior through positive social connections and peer support (Yang et al., 2025), and this evidence does not determine whether baseline support amplifies or attenuates the effects of relational music sharing. Because the study was not powered to detect small interaction effects, the higher-support predictions below (H5 through H8) are treated as exploratory.

Perceived social support refers to the perceived availability of support from significant others, family, and friends (Zimet et al., 1988). In the present model, higher support is expected to make relational cues easier to read as care and to strengthen the associations of the intervention with presence, identity, and prosocial behavior. We hypothesize:

- *H5: The positive association between assignment to the relational-responsive music-sharing intervention bundle and perceived social presence is stronger at higher levels of perceived social support*.
- *H6: The positive association between assignment to the relational-responsive music-sharing intervention bundle and social identity is stronger at higher levels of perceived social support*.
- *H7: The positive association between assignment to the relational-responsive music-sharing intervention bundle and hypothetical third-party charitable allocation is stronger at higher levels of perceived social support*.
- *H8: The indirect associations between assignment to the relational-responsive music-sharing intervention bundle and hypothetical third-party charitable allocation through perceived social presence, social identity, and the serial pathway are stronger at higher levels of perceived social support*.

### 2.5 Beneficiary scope

Finally, theoretical accounts differ in whether the prosocial motivation associated with music-related interaction generalizes across beneficiaries. Relatedness-based reasoning predicts broader other-regarding behavior without specifying a beneficiary (Pavey et al., 2011); identity-based reasoning predicts that social valuation varies with the relevance of a target to a salient identity (Hackel et al., 2017). We therefore test two alternative allocation patterns:

- *H9a: The relational-responsive music-sharing bundle increases allocation to both the music-related and the music-unrelated beneficiary*.
- *H9b: The increase in allocation produced by the relational-responsive music-sharing bundle concentrates on the music-related beneficiary*.

## 3 Methods

### 3.1 Design and participants

This study employed a three-arm randomized field experiment to test hypotheses concerning hypothetical third-party charitable allocation. Willingness to undertake an unpaid helping task was included as a secondary outcome to assess whether any observed effect extended beyond the allocation task and was not an outcome specified in H1. The study also examined whether endpoint associations were compatible with the proposed roles of perceived social presence and social identity and whether those associations varied with perceived social support. The intervention ran for seven consecutive days in participants' own social-media environment, with WeChat Moments as the primary platform; music was routed from commonly used platforms (NetEase Cloud Music, QQ Music) into Moments.

The between-subjects design comprised three arms. In the relational-responsive music-sharing condition (Arm 1), participants shared music with personal, emotional, or relational captions and responded substantively to others' music-related posts. In the informational music posting condition (Arm 2), participants shared music with informational captions, avoiding personal memories, emotional self-disclosure, dedications, and relationship-oriented messages. In the minimal music-use condition (Arm 3), participants used Moments as usual but refrained from posting music-related content during the intervention week.

The focal Arm 1 vs. Arm 2 contrast estimates the joint effect of a relational-responsive music-sharing intervention bundle relative to informational music posting. The bundle simultaneously varied relational and personalized framing, emotional self-disclosure, directed attention, and substantive responsive interaction. The experiment therefore does not identify the contribution of any single component and, because it lacks a relational non-music condition, does not establish that musical content is necessary or uniquely effective. Responsiveness was treated as constitutive of social grooming: Arms 1 and 2 shared the same platform, intervention duration, and assigned daily posting target, and were intentionally differentiated in relational intention, emotional self-disclosure, and responsive attention-giving. As reported below, self-reported posting dosage was equivalent across the two arms; behaviorally recorded posting days were not. The secondary contrast, Arm 2 vs. Arm 3, estimated whether informational posting itself had any effect relative to minimal use.

In the hypothesized model, experimental condition was the independent variable, perceived social presence was the first mediator, social identity was the second mediator, perceived social support was the moderator, and the primary allocation indicator of prosocial behavior was the outcome in the moderated serial mediation analysis of the Arm 1 vs. Arm 2 contrast.

The study was approved by the Academic Ethics Committee of the School of Journalism and Communication at Chongqing University before data collection (approval number CQUSJC2026012). Participants were informed of the study's purpose and general procedure, the voluntary nature of participation, their right to withdraw at any time, and the confidentiality of their responses.

Participants were recruited through a professional survey panel, whose operator handled recruitment, distribution of materials, daily reminders, and initial data collection. The research team retained full control over the study design, randomization procedure, materials, measurements, exclusion criteria, and analysis plan.

Because the intervention required posting on personal accounts, additional privacy protections applied. Participants were instructed not to disclose third-party private information, commercially sensitive information, political content, or other sensitive material. For compliance verification, participants uploaded screenshots of their own music-related posts at the end of the week, after cropping or blurring all third-party names, avatars, and identifiable comments. Screenshots were used only for compliance verification and caption coding, stored separately from survey responses, and deleted after coding.

Participants were recruited from the survey panel's participant pool. Eligibility required (a) being at least 18 years old; (b) being an active WeChat user; (c) using Moments at least 3 days per week; (d) having shared music or encountered others' music sharing during the past month; and (e) passing a baseline attention check. Participants were randomized across the three arms in approximately equal proportions; primary inference concentrated on the focal Arm 1 vs. Arm 2 contrast, with Arm 3 supporting the secondary activity comparison.

Consent, eligibility screening, and the baseline attention check occurred before randomization. The retained dataset begins with 583 randomized participants (Arm 1, *n* = 195; Arm 2, *n* = 194; Arm 3, *n* = 194). After randomization, 15 participants failed the endpoint attention check (Arm 1, *n* = 5; Arm 2, *n* = 7; Arm 3, *n* = 3). No participant withdrew, no endpoint survey was incomplete, and no duplicate-response exclusion was applied after randomization. The resulting modified intention-to-treat (mITT) sample comprised 568 participants (Arm 1, *n* = 190; Arm 2, *n* = 187; Arm 3, *n* = 191), including 377 participants in the focal Arm 1 vs. Arm 2 analysis.

Complete endpoint data, including the outcome, mediator, and covariate measures, were available for all 583 randomized participants, including the 15 who failed the endpoint attention check. Accordingly, all randomized participants were additionally retained in their assigned conditions for a full intention-to-treat robustness analysis (Arm 1, *n* = 195; Arm 2, *n* = 194; Arm 3, *n* = 194; focal Arm 1 vs. Arm 2 sample, *n* = 389).

Within the mITT sample, 50.9% identified as female, 47.2% as male, and 1.9% as another gender or preferred not to answer; ages ranged from 18 to 38 years (*M* = 25.0, SD = 4.5). Educational backgrounds comprised bachelor's degrees (59.2%), master's degrees (20.1%), junior college (11.3%), high school or below (7.0%), and doctoral degrees or above (2.5%). Participants were distributed across first-tier (26.8%), new first- and second-tier (47.0%), and third-tier-or-below (26.2%) cities. A sensitivity analysis indicates that the focal subsample affords 80% power (α = 0.05, two-tailed) to detect effects of d ≥0.29 on the primary contrast; interaction and moderated-indirect tests were treated as exploratory.

### 3.2 Procedure and conditions

The study had four stages: baseline survey, random assignment, 7-day field intervention, and endpoint survey with behavioral outcome measurement.

#### 3.2.1 Baseline survey

Participants provided informed consent and completed eligibility items, demographics, baseline music-sharing frequency, perceived social support, and baseline trait prosociality. Perceived social support was measured pre-intervention because it served as the moderator; baseline trait prosociality was measured as a precision covariate. Random assignment. Eligible participants were randomized by computer after the baseline survey; baseline balance across arms was examined empirically (Section 4.2).

#### 3.2.2 Seven-day intervention

Each participant received an instruction card for the assigned condition. Arm 1 posted at least one music item daily with a personal, emotional, or relational caption and responded substantively to at least one music-related post daily. Arm 2 posted at least one music item daily with an informational caption and maintained ordinary baseline interaction without adding relational responses for the study. Arm 3 used Moments normally but posted no music-related content. Each evening, participants completed a 2-minute micro-log recording whether they had posted, what they had shared, the exact caption, whether they had responded to others' music posts, and any difficulty completing the task. These logs served as compliance indicators and as behavioral data for coding the relational, emotional, informational, and responsive properties of participants' sharing.

#### 3.2.3 Endpoint survey (Day 8)

The survey was administered in a fixed order: manipulation checks, perceived social presence, social identity, state affect, feedback received, a study-complete transition page, the behavioral allocation task, the helping request, suspicion probe, interference check, and debriefing. This order protected the focal mediators from affect-measurement priming while still capturing state affect before the behavioral outcome. The allocation task was presented as a separate module to reduce demand characteristics.

#### 3.2.4 Relational-responsive music sharing (Arm 1)

For 7 days, participants shared at least one music-related post each day, such as a song, playlist, lyric, video, or music-related memory. Each post included a personal, emotional, or relational caption connecting the music to a memory, emotion, person, relationship, or shared experience. Participants also responded substantively to at least one music-related post each day through a comment or reply that went beyond a bare like. An example caption was: “This song reminds me of the nights we stayed up together before graduation. I still think of you all whenever I hear it.” This condition operationalized the relational-responsive music-sharing intervention bundle through music-related self-disclosure, emotional expression, directed attention, and reciprocal interaction.

#### 3.2.5 Informational music posting (Arm 2)

Participants also shared at least one music-related post daily for 7 days, but captions were informational (song facts, genre, artist, chart performance, style, rhythm, release year, or other objective description), explicitly excluding personal memories, emotional self-disclosure, dedications, and relationship-oriented messages. An example caption: “This track was released in 1997 and uses a five-note pentatonic hook. It re-entered the charts this year.” Arms 1 and 2 shared the same platform, intervention duration, and assigned daily posting target. Because participants selected their own music, however, the musical materials themselves were neither identical nor prospectively matched across conditions. The two arms differed on relational responsiveness, which formed part of the treatment.

#### 3.2.6 Minimal music-use (Arm 3)

**Figure 1:**
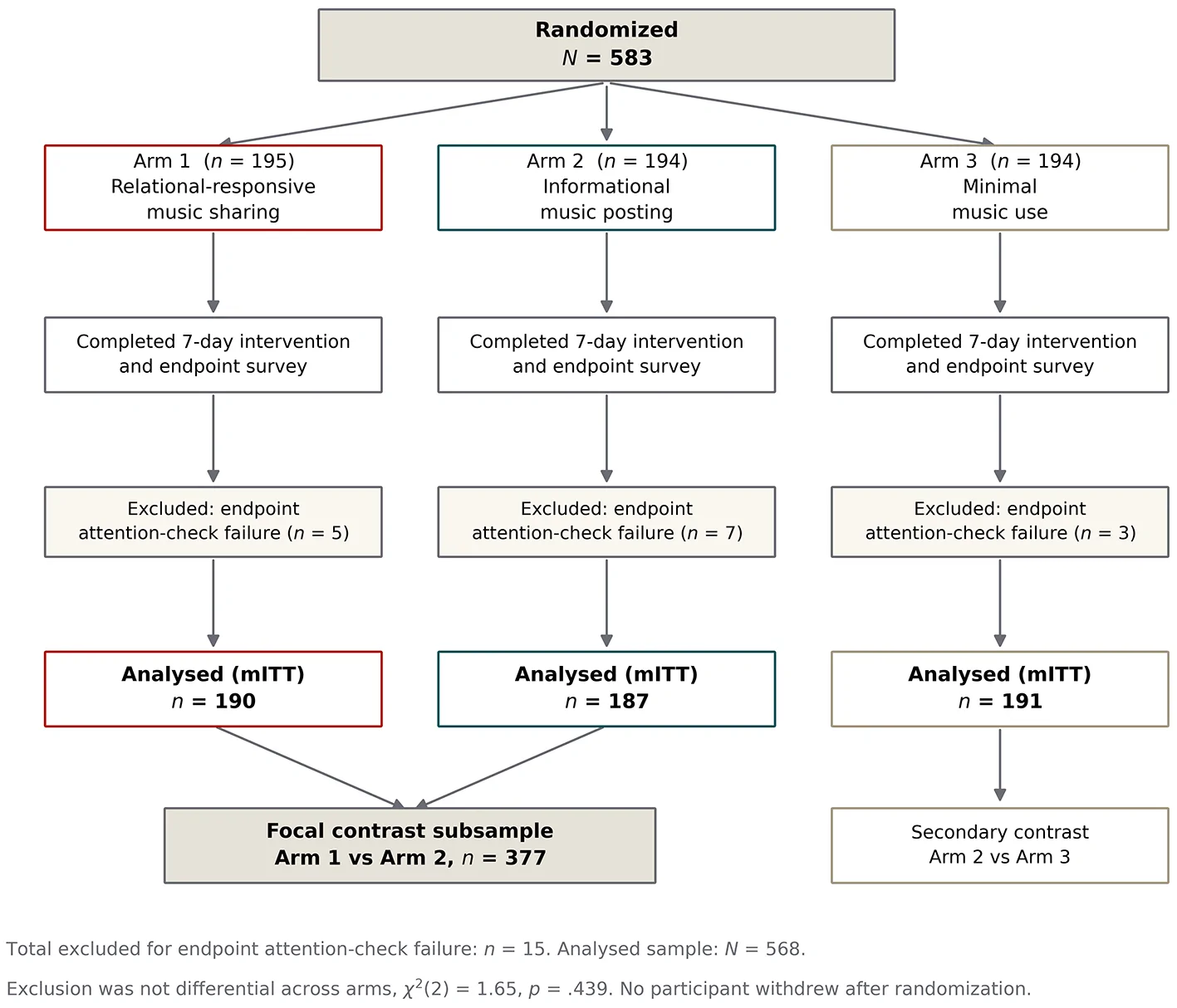
Participant flow from randomization to the mITT analysis. All 583 randomized participants were retained in the full intention-to-treat robustness analysis.

Participants used Moments as usual but posted no music-related content during the week, browsing normally and maintaining ordinary interaction habits. This provided a baseline for the effect of informational posting relative to minimal use. Figure 1 shows the participant flow for this study.

### 3.3 Measurement

All questionnaire measures were administered in Chinese. Unless otherwise stated, participants responded on a five-point Likert scale (1 = strongly disagree to 5 = strongly agree). For scales developed in English, two bilingual researchers independently translated and contextually adapted the items into Chinese. They compared their versions, reconciled discrepancies against the source wording, and agreed on a common administration version. This translation procedure is separate from the derivation of the behavioral coding variables described below. Because the study was set in a music-sharing context, items were minimally adapted to reference “this week's music-sharing interactions” or “music-related posts” where appropriate; these changes contextualized the measures without altering the core conceptual meaning of the original scales.

The primary hypothesized outcome was hypothetical third-party charitable allocation under a fixed budget, designated primary because it imposed a trade-off against self-interest: every token allocated was a token not retained. A secondary outcome assessed willingness to complete an unpaid helping task, which carried a cost in time and effort but not in foregone resources; this secondary measure was used to examine whether the allocation result generalized to another prosocial outcome. Neither indicator is equated with prosocial behavior in general.

The manipulation checks assessed whether the manipulation differentiated posting dosage, relational intention, and responsive interaction across conditions. Item design followed the operational logic of social grooming research, in which grooming is reflected in purposeful self-disclosure, relational maintenance, and responsive interaction, none of which posting frequency alone captures, and in which posting, liking, and commenting are treated as relational acts through which users signal attention and maintain ties (Lin, 2019; Wang and Wang, 2025).

Two layers were used. The frequency layer asked whether participants shared or reposted music content, posted music-related updates, and increased their music posting during the week; the expected pattern was Arm 1 ≈ Arm 2 > Arm 3. The relational-intention layer asked whether participants' music posts expressed feelings or maintained relationships, whether their sharing was directed at particular people or relationships, whether they engaged in back-and-forth interaction, and whether they responded substantively to others' posts; the expected pattern was Arm 1 > Arm 2 ≈ Arm 3. This pattern reflects the definition of the relational-responsive music-sharing intervention bundle, in which responsive attention-giving is constitutive of the treatment; it is a designed feature of the manipulation.

Beyond self-report, captions recorded in the daily logs and verified against privacy-screened screenshots were coded for relationality (references to particular people, relationships, shared memories, care, gratitude, longing, encouragement, or closeness), emotionality (explicit affective expression), informationality (factual description of the music), and responsiveness (no response, a minimal response such as a like, or a substantive comment or reply). Coding was conducted independently by two trained coders who were not provided with participants' randomized condition labels. Because condition-specific instructions could nevertheless be inferred from the content of the posts, this procedure is not treated as successful masking of condition assignment. The coders worked independently and resolved disagreements through discussion before codes were aggregated to the participant level. A formal intercoder reliability coefficient was not computed at the time of coding and cannot be reconstructed because rater-level records were not retained. The resulting coded indicators are therefore treated as complementary behavioral evidence and interpreted jointly with the self-reported manipulation checks, rather than as a stand-alone validation of the manipulation.

Perceived social presence was adapted from the Networked Minds measure (Harms and Biocca, 2004; Biocca et al., 2003), which conceptualizes social presence as the degree to which people feel mutually aware of and psychologically involved with one another in mediated interaction. The present study focused on the components central to music sharing, perceived co-presence and mutual attentional awareness. Referencing their music-sharing interactions during the past week, participants rated items such as “During this week's music-sharing interactions, I felt that the people I interacted with were really ‘there' with me,” “The presence of the people I interacted with was obvious to me,” “I felt that they noticed me,” and “I felt that they were paying attention to me.” The Networked Minds measure was preferred over platform-oriented social presence scales because the model requires a psychological perception that others are socially present and mutually engaged, not perceived interface affordance or media richness. Items were adapted from the co-presence and attentional-allocation dimensions of the measure, and the original seven-point response format was changed to a five-point format.

Social identity was adapted from Lee et al. (2011) study of music-sharing behavior on social network services, which identified social identity and social presence as predictors of music-sharing intention. Here, it referred to participants' perceived belonging, closeness, and positive identification with the people involved in their music-sharing interactions. To avoid a diffuse referent, all items used the same concrete anchor, “the people I came into contact with in this week's music-sharing interactions,” for example, “I feel a sense of belonging with the people I came into contact with in this week's music-sharing interactions,” and “I feel togetherness or closeness with [them].” This referent distinguished social identity (belonging or “we-ness” with those encountered) from perceived social presence (perceiving others as present and attentive during the interaction). The referent was changed to the people encountered during this week's music-sharing interactions, and the original seven-point response format was changed to a five-point format.

Perceived social support was measured at baseline with the Multidimensional Scale of Perceived Social Support (MSPSS; Zimet et al., 1988), a 12-item measure of perceived support adequacy from family, friends, and significant others, rated on the original seven-point scale (1 = very strongly disagree to 7 = very strongly agree). Sample items: “There is a special person who is around when I am in need”; “My family really tries to help me”; “I can count on my friends when things go wrong.” The overall mean served as the main moderator; the friends subscale was used in robustness analyses because grooming on Moments occurs primarily within peer and friend networks. The MSPSS captures the perceived availability of support, which keeps it conceptually distinct from the prosocial-behavior outcome.

Baseline trait prosociality was measured with the Prosocialness Scale for Adults (Caprara et al., 2005), 16 items capturing helping, sharing, caring, and empathic responding (e.g., “I try to help others”; “I try to console those who are sad”). Measured only at baseline and entered as a covariate, not as the dependent variable, it improved precision and accounted for stable individual differences in prosocial disposition, consistent with the aim of testing whether the intervention affected behavioral prosocial allocation.

State affect was measured at endpoint with the International Positive and Negative Affect Schedule Short Form (I-PANAS-SF; Thompson, 2007). Rating how they felt “right now,” participants responded to positive adjectives (active, determined, attentive, inspired, alert) and negative adjectives (upset, hostile, ashamed, nervous, afraid), scored as separate five-item means. Affect was included as a rival mechanism, not a focal mediator: relational sharing may shift immediate mood, and measuring affect allowed the study to test whether the presence and identity pathways held beyond a mood-based explanation. It was measured after the focal mediators but before the allocation task, so affect was not contaminated by warm glow or regret following the allocation.

Participants reported the approximate number of likes and comments their music posts received during the week and rated whether that feedback was positive and supportive. Feedback was not treated as a focal mediator because it is endogenous to the condition: relational-responsive sharing may naturally elicit more responses, and controlling for that feedback in the main model could remove part of the treatment process. It was used in robustness analyses to test whether the presence and identity paths remained stable when the received social reward was taken into account.

The primary dependent variable was a fixed-budget allocation task presented in a separate allocation module. Participants were asked to imagine that they held 10 tokens, each carrying a stated value of 1 RMB, and to divide them among themselves, a rural music-education public-interest program, and a general poverty-relief charity, with the three entries required to sum to 10. The stated token value was provided to make the magnitude of the trade-off concrete. The task was simulated: the tokens carried no implemented monetary conversion, were not added to participant compensation, and generated no transfer to either beneficiary. The allocation rule and the fixed budget were shown before the decision screen. The primary outcome was the total number of tokens allocated to the two charitable beneficiaries (0–10), capturing the self-vs.-other trade-off under a fixed budget: more allocated meant fewer retained. The task is therefore a stated allocation decision under a fixed budget, not an incentivized transfer, and it retains the trade-off structure that distinguishes allocation measures from agreement-rated helping intentions. The two destinations tested beneficiary scope, music-education allocations representing music-related giving, and poverty-relief allocations representing giving to a music-unrelated beneficiary. Because both drew on the same endowment, the destination-specific variables were not treated as independent primary outcomes; the split was used to examine whether the effect generalized beyond the music domain or concentrated in the music-related beneficiary.

After the allocation task, participants were asked whether they would spend an additional 3 min helping evaluate short materials for another task, described as voluntary and payment-irrelevant. Willingness was recorded as binary, and the number of materials completed as an additional indicator. This provided a non-monetary prosocial measure: willingness to sacrifice time and effort.

### 3.4 Analysis

Several procedures protected data quality: attention-check items embedded in the baseline and endpoint surveys; daily micro-logs assessing compliance; screenshots verifying the presence and style of posts; and screening of completion time, duplicate responses, and inconsistent answers to identify careless or invalid records.

The primary analysis was conducted on the modified intention-to-treat (mITT) sample: all randomized participants who passed the endpoint attention check. Per-protocol analyses were conducted as sensitivity checks on the focal Arm 1 vs. Arm 2 subsample. Participants in Arm 1 were per-protocol compliant if they posted music content on at least five of 7 days, had captions coded relational on at least half of their posting days, and responded to others' music posts on at least four of 7 days. Participants in Arm 2 were compliant if they posted on at least five of 7 days and their captions had a mean informationality of at least 1.0 on the 0–2 coder scale.

As an additional robustness check, the primary allocation model, secondary helping model, and moderated serial mediation specification were re-estimated in a full intention-to-treat sample retaining every randomized participant in the assigned condition, irrespective of endpoint attention-check performance. No outcome imputation was required because endpoint data were complete.

No post-randomization exclusions were applied other than the endpoint attention check; no participant withdrew, no endpoint survey was incomplete, and no duplicate or invalid records were removed. The baseline attention check formed part of the eligibility screening before randomization. Participants who correctly identified the core hypothesis in the suspicion probe were retained in the primary analysis and excluded only in the corresponding sensitivity analysis, as were participants who reported knowing another participant and seeing that participant's intervention posts.

Descriptive statistics were computed for all variables. Exclusion rates were compared across arms with a chi-square test. Baseline equivalence was assessed with ANOVA for continuous variables and chi-square tests for categorical variables.

Reliability was evaluated with Cronbach's alpha and composite reliability (Supplementary material). Confirmatory factor analysis examined the six-factor measurement structure comprising perceived social presence, social identity, perceived social support, trait prosociality, positive affect, and negative affect, together with a focused two-factor comparison of the nine perceived social presence and social identity items. Convergent validity was assessed via factor loadings and average variance extracted; discriminant validity via the Fornell–Larcker criterion, the focused confirmatory comparison, and the heterotrait–monotrait ratio (Henseler et al., 2015); multicollinearity via variance inflation factors; and common method variance via Harman's single-factor test and the one-factor confirmatory model. Neither common-method diagnostic was treated as establishing the absence of method variance.

Manipulation checks were analyzed with one-way ANOVAs and planned contrasts. Expected patterns were Arm 1 ≈ Arm 2 > Arm 3 for posting frequency; Arm 1 > Arm 2 ≈ Arm 3 for relationality, emotionality, and responsiveness; and Arm 2 > Arm 1 for informationality. Coded caption data supplemented the self-reports.

The primary model was a moderated serial mediation specification, estimated on the focal Arm 1 vs. Arm 2 subsample. Condition was coded 1 (relational-responsive bundle) vs. 0 (informational music posting); perceived social presence entered as M1, social identity as M2, perceived social support as the exploratory moderator, and total allocated tokens as *Y*, with baseline trait prosociality, baseline music-sharing frequency, age, and gender as covariates. Bootstrap analyses (10,000 resamples) estimated indirect associations, conditional indirect associations, and indices of moderated mediation; an interval excluding zero was treated as statistical support for the corresponding association.

Secondary analyses compared Arm 2 and Arm 3 to estimate the effect of informational posting relative to minimal use. The beneficiary-scope hypothesis was tested by regressing the within-participant allocation difference (music-education tokens minus poverty-relief tokens) on the condition. With two repeated destinations, this difference-score model is algebraically equivalent to the condition-by-beneficiary interaction and directly estimates whether the condition effect differed across beneficiaries.

Robustness analyses re-estimated the primary model (a) on the per-protocol sample (b) excluding suspicion-flagged cases (c) excluding interference-positive cases (d) using the MSPSS friends subscale as an alternative moderator, and (e) adding state affect and feedback received as rival mechanisms, testing whether the presence and identity pathways held beyond mood and received social reward. Because total allocated tokens are an integer bounded between 0 and 10, two sensitivity models were estimated alongside the linear specification. The outcome was rescaled to the unit interval by dividing by 10, and a fractional logit was fitted by quasi-maximum likelihood with a logit link and robust standard errors, with the average marginal effect converted back to the token scale (Papke and Wooldridge, 1996); a proportional-odds logit and a distribution-free Mann–Whitney test were estimated as further checks. Point estimates and 95% confidence intervals are reported for every robustness analysis, including those that are not statistically significant.

All statistical analyses were conducted in Python, with the moderated serial mediation model implemented as a system of ordinary-least-squares equations and 10,000 bootstrap resamples; publication figures were produced with Matplotlib.

Because perceived social presence and social identity were measured in the same endpoint survey and neither mediator was randomized, indirect effects are reported throughout as associational paths consistent with the proposed presence-to-identity ordering. Random assignment identifies the effect of condition on the measured outcomes, but it does not randomize the mediators or identify their temporal order (Bullock et al., 2010; Pirlott and MacKinnon, 2016). The data contain no temporal information that would order the two mediators, and the observed pattern is equally compatible with the reverse ordering and with a parallel structure in which a third factor drives both.

## 4 Results

### 4.1 Randomization, manipulation, and measurement

Following the analysis plan set out above, primary inference rested on the focal Arm 1 vs. Arm 2 contrast, with Arm 3 supporting the secondary comparison; results are reported in the order in which the hypotheses were introduced.

Of the 583 randomized participants, 568 passed the endpoint attention check and constituted the modified intention-to-treat (mITT) sample. The focal Arm 1 vs. Arm 2 subsample numbered 377. The 15 cases were excluded only from the primary mITT analysis. Endpoint attention-check failure was small (2.6%) and not differential across arms, $\chi_{\left(2\right)}^{2}$ = 1.65, *p* = 0.439. No participant withdrew or had incomplete endpoint data. Because complete outcome data remained available, all 583 randomized participants were retained in the full intention-to-treat robustness analysis.

Randomization produced well-balanced conditions. One-way analyses of variance across the three arms were non-significant for every baseline covariate: age, *F*_(2, 565)_ = 0.38, *p* = 0.681; baseline music-sharing, *F* = 0.49, *p* = 0.611; perceived social support, *F* = 0.37, *p* = 0.694; trait prosociality, *F* = 1.13, *p* = 0.323; and baseline WeChat use, *F* = 0.52, p = 0.596. Standardized mean differences between the focal arms were correspondingly small (all |*d*| ≤ 0.15), and gender was evenly distributed across arms, $\chi_{\left(4\right)}^{2}$ = 1.04, *p* = 0.904. Baseline trait prosociality, baseline music-sharing frequency, age, and gender were retained as covariates in all outcome models, consistent with the analytic plan, to absorb residual individual differences and improve precision.

**Figure 2:**
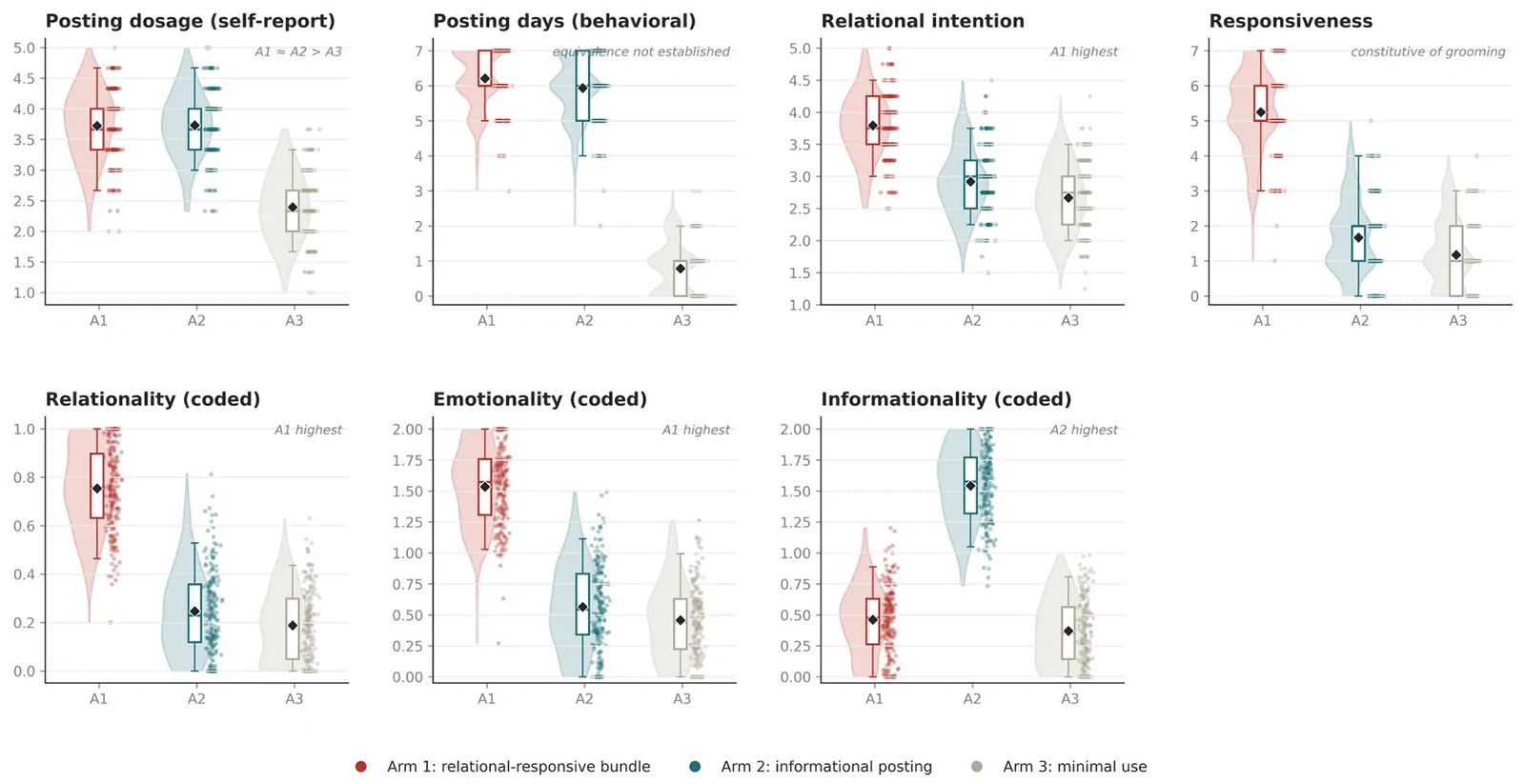
Manipulation-fidelity indicators across the three conditions.

The manipulation differentiated the two active arms on the relational-responsive components of the bundle (Figure 2; Table 1). The arms shared the same assigned daily posting target, and self-reported posting dosage was equivalent within the analysis bounds of *d* = ±0.30 (*M* = 3.72 vs. 3.73, *d* = −0.01, TOST *p* = 0.003). Behaviorally recorded posting days were higher in Arm 1 than in Arm 2 (*M* = 6.21 vs. 5.94, *d* = 0.31); equivalence was not established (TOST *p* = 0.554), and the difference was non-zero, *t*_(375)_ = 3.05, *p* = 0.002. Thus, the active conditions are not described as fully dosage matched. Adding behavioral posting days to the primary model left the condition estimate similar, *b* = 0.778, SE = 0.194, 95% CI [0.396, 1.160], *p* < 0.001; posting days was not associated with allocation in that model, *b* = −0.076, 95% CI [−0.286, 0.133], *p* = 0.474. Arm 3 posted on an average of 0.79 days.

**Table 1:** Manipulation-check means (SD) and planned contrasts by arm.

Measure	Arm 1	Arm 2	Arm 3	A1 vs. A2 (*d*)	A2 vs. A3 (*p*)
Posting dosage (self-report)	3.72 (0.58)	3.73 (0.54)	2.40 (0.56)	−0.01	< 0.001
Posting days (behavioral)	6.21 (0.83)	5.94 (0.91)	0.79 (0.75)	+0.31	< 0.001
Relational intention	3.79 (0.51)	2.92 (0.53)	2.67 (0.52)	+1.69	< 0.001
Relationality (coded)	0.75 (0.18)	0.25 (0.17)	0.19 (0.15)	+2.91	< 0.001
Emotionality (coded)	1.54 (0.32)	0.56 (0.34)	0.46 (0.30)	+2.92	0.001
Responsiveness (days)	5.25 (1.13)	1.67 (1.06)	1.17 (0.99)	+3.27	< 0.001
Informationality (coded)	0.46 (0.27)	1.54 (0.30)	0.37 (0.26)	−3.80	< 0.001

Equivalence between Arms 1 and 2 was established for self-reported posting dosage by two one-sided tests (bounds *d* = ±0.30, *p* = 0.003). Behavioral posting days show *d* = 0.31, outside the same bounds; equivalence is not established for this indicator. All other Arm 1 vs. Arm 2 contrasts were significant at *p* < 0.001 and were differentiated by design.

Despite the small difference in behavioral posting days, the relational-responsive content of the two active arms diverged clearly, as the design intended. Independently coded relationality was higher in Arm 1 than Arm 2 (proportion 0.75 vs 0.0.25), *t*_(375)_ = 28.3, *p* < 0.001, *d* = 2.91; coded emotionality was higher (*M* = 1.54 vs. 0.56), *d* = 2.92; and responsive engagement was greater (5.25 vs. 1.67 response-days), *d* = 3.27. Informationality reversed as intended, being higher in Arm 2 (*M* = 1.54 vs. 0.46), *d* = −3.80. Self-reported relational intention mirrored the coded indicators (Arm 1 *M* = 3.79 vs. Arm 2 *M* = 2.92, *d* = 1.69), and Arm 2 was close to Arm 3 on relational intention (*M* = 2.92 vs. 2.67), consistent with informational posting adding little relational meaning relative to minimal use. The independently coded behavioral indicators and the self-reported manipulation checks therefore converged in direction, although the coded indicators should be interpreted cautiously given the unavailable intercoder-reliability estimate.

The manipulation, therefore, differentiated the relational-responsive bundle while holding the assigned daily target constant, not while establishing equivalence on every realized dosage indicator. Self-reported posting dosage was equivalent, but behavioral posting days were not. Responsiveness was a constitutive treatment component, not a covariate to be partialled out. Figure 2 displays the manipulation-fidelity results.

All multi-item scales showed acceptable-to-strong internal consistency (Table 2). Cronbach's α ranged from 0.706 to 0.920, and composite reliability estimated from the confirmatory factor model ranged from 0.707 to 0.921. Average variance extracted was 0.427 for perceived social presence, 0.461 for social identity, 0.491 for perceived social support, 0.369 for trait prosociality, 0.325 for positive affect, and 0.437 for negative affect; every value fell below the conventional 0.50 criterion, and convergent validity is therefore treated as limited. The six-factor measurement model fit the data well, $\chi_{\left(1019\right)}^{2}$ = 1141.89, CFI = 0.986, TLI = 0.985, RMSEA = 0.015; a one-factor model fit poorly, $\chi_{\left(1,034\right)}^{2}$ = 6034.92, CFI = 0.426, TLI = 0.400, RMSEA = 0.092.

**Table 2:** Reliability and validity of measures.

Construct	Items	α	CR	AVE
Perceived social presence (M1)	5	0.788	0.789	0.427
Social identity (M2)	4	0.774	0.774	0.461
Perceived social support (W)	12	0.920	0.921	0.491
Trait prosociality (covariate)	16	0.903	0.903	0.369
Positive affect	5	0.706	0.707	0.325
Negative affect	5	0.795	0.795	0.437

α, Cronbach's alpha; CR, composite reliability; AVE, average variance extracted. Discriminant validity for the focal mediators was assessed using the focused CFA comparison and HTMT; see the text and Supplementary material.

Discriminant validity was adequate. The maximum heterotrait-monotrait (HTMT) ratio among the six constructs was 0.50, well below the conservative 0.85 threshold, indicating that perceived social presence, social identity, perceived social support, and affect were empirically distinct. The Fornell–Larcker criterion was likewise satisfied: the square root of average variance extracted for each construct exceeded that construct's correlations with every other construct (Supplementary Table S20). This addresses the central construct-separation concern for this design, that a set of positively valenced social constructs might collapse into a single “positive social connection” factor; the one-factor confirmatory model reported above is the formal test of that possibility, and it does not fit. For the nine presence and identity items specifically, a two-factor model fit well, $\chi_{\left(26\right)}^{2}$ = 35.41, CFI = 0.993, RMSEA = 0.025, and fit better than a one-factor model, $\chi_{\left(27\right)}^{2}$ = 353.19, CFI = 0.759, RMSEA = 0.146, Δ$\chi_{\left(1\right)}^{2}$ = 317.78, *p* < 0.001. The two focal mediators were moderately correlated (*r* = 0.39), indicating that the two constructs are related but not redundant; the correlation does not establish their temporal ordering.

### 4.2 Primary effect and serial indirect associations

**Figure 3:**
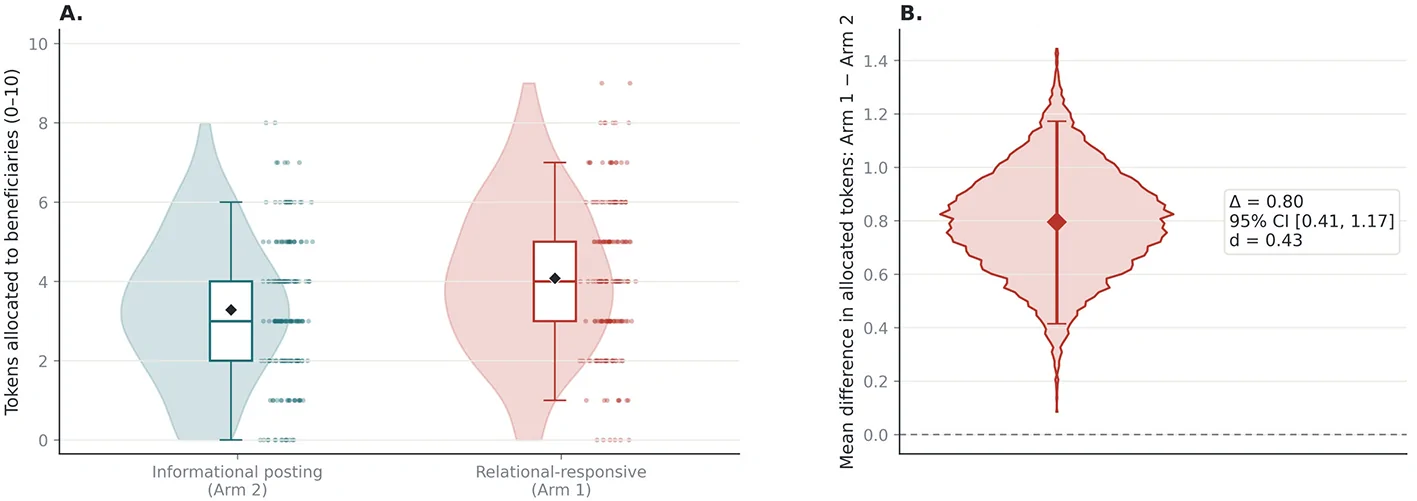
Adjusted effect of the relational-responsive bundle on total hypothetical charitable allocation. **(A)** Raw distributions. **(B)** Bootstrap mean difference.

H1 was supported: assignment to the relational-responsive music-sharing intervention bundle increased total hypothetical third-party charitable allocation relative to informational music posting. Participants in Arm 1 allocated a mean of 4.08 tokens (S*D* = 1.94) of their 10-token budget, whereas participants in Arm 2 allocated a mean of 3.28 tokens (S*D* = 1.82). In the covariate-adjusted ordinary least squares model with HC3 heteroskedasticity-consistent standard errors, the effect of condition was *b* = 0.76, SE = 0.19, *t*_(371)_ = 3.91, *p* < 0.001, 95% CI [0.38, 1.14], corresponding to a medium effect (*d* = 0.43). The bootstrap mean difference was 0.80, 95% CI [0.41, 1.17] (Figure 3).

The secondary contrast was null, and the estimate is reported in full: informational music posting did not differ from minimal music use on total allocation (*M* = 3.28 vs. 3.39), *b* = −0.08, SE = 0.19, 95% CI [−0.46, 0.29], *p* = 0.668, *d* = −0.06. A *post hoc* equivalence test against bounds of *d* = ±0.30 was consistent with equivalence, *p* = 0.010; because these bounds were prespecified for the dosage manipulation check, not for this outcome comparison, the test is reported as a sensitivity analysis and not as a prespecified equivalence result. Informational music posting did not produce a detectable difference from minimal music use in this task. This comparison does not establish that music exposure on its own is generally ineffective.

**Figure 4:**
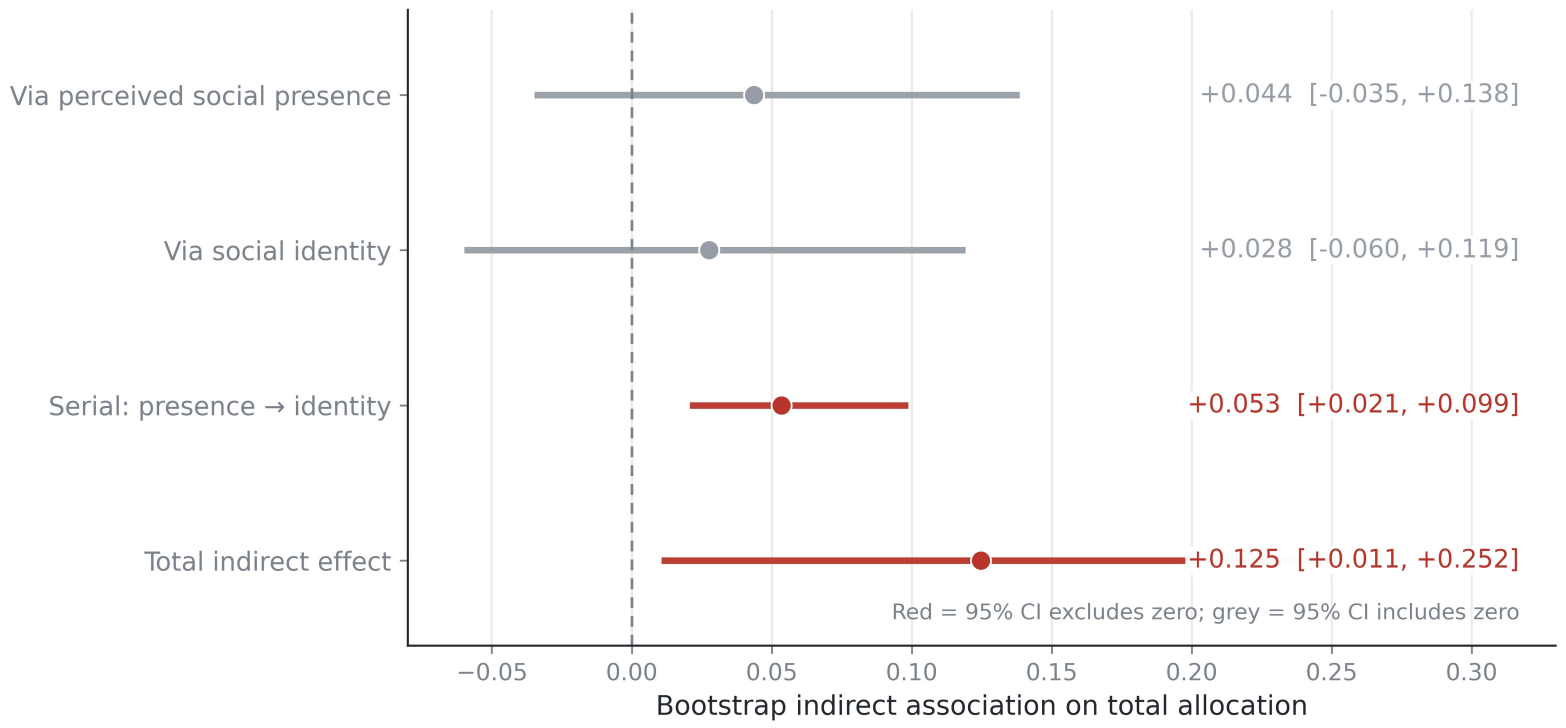
Associational indirect pathways linking the condition to the total hypothetical charitable allocation.

The moderated serial mediation model is summarized in Table 3, and the bootstrap indirect effects are displayed in Figure 4. Because both mediators were measured on the same endpoint occasion and neither was manipulated, the indirect effects are reported as associational paths consistent with the proposed presence-to-identity ordering. The ordering is an assumption of the model, not a finding of the study.

**Table 3:** Moderated serial mediation model.

Panel A. Path coefficients				
Path	Estimate	SE	95% CI	p
X → M1 (a1)	0.269	0.067	[0.137,0.401]	< 0.001
W → M1 (a2)	0.109	0.055	[0.001,0.218]	0.049
X × W → M1 (a3)	0.119	0.077	[−0.032,0.270]	0.121
M1 → M2 (d21)	0.341	0.056	[0.231,0.451]	< 0.001
X → M2 (a4)	0.047	0.074	[−0.097,0.192]	0.520
W → M2 (a5)	0.092	0.059	[−0.025,0.209]	0.121
X × W → M2 (a6)	0.124	0.083	[−0.038,0.286]	0.133
M1 → Y (b1)	0.162	0.150	[−0.132,0.457]	0.279
M2 → Y (b2)	0.582	0.133	[0.320,0.844]	< 0.001
X → Y, direct (c′)	0.622	0.188	[0.252,0.992]	0.001
W → Y (b3)	0.120	0.153	[−0.180,0.420]	0.431
X × W → Y (c3)	0.223	0.211	[−0.192,0.639]	0.291
Panel B. Indirect associations				
Association			Estimate	95% bootstrap CI
Via perceived social presence			0.044	[−0.035, 0.138]
Via social identity			0.028	[−0.060, 0.119]
Serial: presence → identity			0.053	[0.021, 0.099]
Total indirect association			0.125	[0.011, 0.252]
Panel C. Conditional direct associations				
Perceived social support			Estimate	95% bootstrap CI
−1 SD			0.427	[−0.057, 0.913]
Mean			0.622	[0.265, 0.988]
+1 SD			0.817	[0.316, 1.323]
Panel D. Indices of moderated mediation				
Index			Estimate	95% bootstrap CI
Via perceived social presence			0.019	[−0.017, 0.077]
Via social identity			0.072	[−0.014, 0.177]
Via serial pathway			0.024	[−0.005, 0.060]

M1, perceived social presence; M2, social identity, referenced to the people encountered in the week's music-sharing interactions; W, mean-centered perceived social support; Y, total hypothetical tokens allocated to the two charitable beneficiaries. Covariates: baseline trait prosociality, baseline music-sharing frequency, age, and gender. Path-coefficient confidence intervals use HC3 standard errors; indirect, conditional, and index confidence intervals are 10,000-resample percentile bootstrap intervals.

Mediator pathways. Condition predicted perceived social presence, *a*_1_ = 0.27, 95% CI [0.14, 0.40], p < 0.001: the relational-responsive condition raised presence relative to informational posting. Perceived social presence was positively associated with social identity, *d*_21_ = 0.34, 95% CI [0.23, 0.45], *p* < 0.001, consistent with the specified presence-before-identity ordering. Social identity was positively associated with allocation, *b*_2_ = 0.58, 95% CI [0.32, 0.84], *p* < 0.001; the presence-to-allocation coefficient was not statistically significant, *b*_1_ = 0.16, 95% CI [−0.13, 0.46], *p* = 0.28. Within the specified model, this pattern was compatible with an indirect association involving both mediators. The direct association between condition and allocation remained statistically significant with both mediators in the model, *c*′ = 0.62, 95% CI [0.25, 0.99], *p* = 0.001.

The data were compatible with H4′s proposed serial indirect association. The serially specified path from condition through perceived social presence and social identity to allocation was 0.053, 95% bootstrap CI [0.021, 0.099]. A reverse-order sensitivity path from condition through identity and presence to allocation was 0.006, 95% bootstrap CI [−0.005, 0.023]. This comparison does not constitute a temporal test because concurrent measurement prevents causal ordering.

H2 and H3 were not supported. The presence-only indirect association was 0.044, 95% bootstrap CI [−0.035, 0.138], and the identity-only indirect association was 0.028, 95% bootstrap CI [−0.060, 0.119]; both intervals included zero. The serial product specified in H4 nevertheless excluded zero. Given the concurrent assessment of the mediators, this result is interpreted as an endpoint association compatible with the proposed ordering, not as evidence of a causal mediation process.

### 4.3 Moderation and Beneficiary Scope

The exploratory moderation hypotheses were not supported (Table 3). Sample-size planning targeted the primary Arm 1 vs. Arm 2 contrast and did not establish power for interactions or moderated indirect associations, so H5 through H8 are reported here, as in the Hypothesis Development, as exploratory. The condition with support interaction was non-significant in every equation: for the effect on perceived social presence, *a*_3_ = 0.12, *p* = 0.12 (H5 not supported); for the effect on social identity, *a*_6_ = 0.12, *p* = 0.13 (H6 not supported); and for the direct effect on allocation, *c*_3_ = 0.22, *p* = 0.29 (H7 not supported). Conditional effects estimated at low, mean, and high support were similar in magnitude, and none provided evidence of moderation (Table 3).

All three indices of moderated mediation included zero: via presence, 0.019, 95% CI [−0.017, 0.077]; via identity, 0.072, 95% CI [−0.014, 0.177]; and via the serial pathway, 0.024, 95% CI [−0.005, 0.060] (H8 not supported). Because these tests were exploratory and relatively underpowered, the null results provide no evidence of moderated mediation and do not establish that moderation is absent.

**Figure 5:**
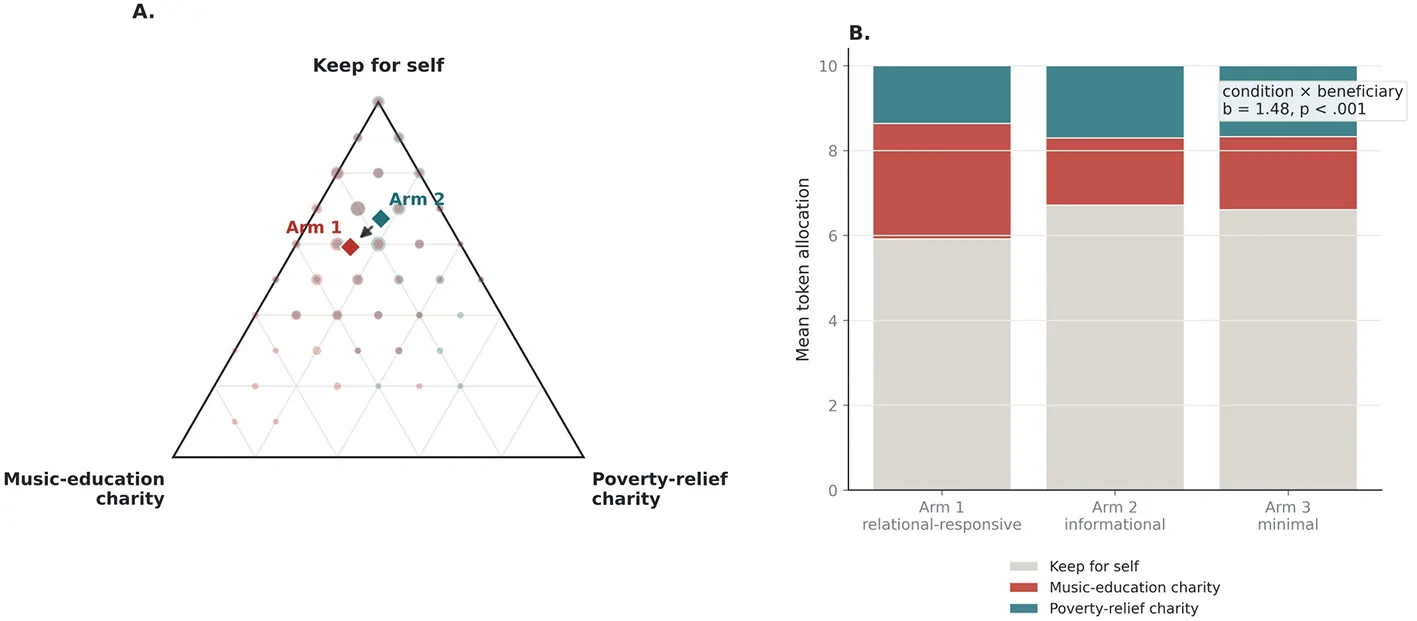
Total and beneficiary-specific allocation under the fixed 10-token budget. **(A)** Allocation geometry. **(B)** Mean allocation by arm.

The beneficiary-scope test distinguished a generalized from a domain-specific pattern of allocation (Figure 5; Table 4). Because the two beneficiary amounts came from the same fixed 10-token budget, the destination-specific contrast was estimated by regressing the within-participant difference (music-education minus poverty-relief tokens) on condition. This model is algebraically equivalent to the condition-by-beneficiary interaction for two repeated destinations and avoids a random-intercept specification that is not identified for a fixed-sum outcome, since the two amounts are negatively correlated within a person. The condition effect on the difference score was *b* = 1.481, SE = 0.196, *t*_(375)_ = 7.55, 95% CI [1.095, 1.866], *p* < 0.001. Decomposed by destination, the relational-responsive bundle increased allocation to the music-education cause (Arm 1 *M* = 2.72 vs. Arm 2 *M* = 1.58, *d* = 0.78, *p* < 0.001) but did not increase, and in fact slightly reduced, allocation to the music-unrelated poverty-relief charity (*M* = 1.36 vs. 1.70, *d* = −0.28, *p* = 0.006).

**Table 4:** Beneficiary-scope analysis: allocation by condition and destination.

Beneficiary	Arm 1 M	Arm 2 M	*d*	*p*
Music-education charity	2.72	1.58	+0.78	< 0.001
Poverty-relief charity	1.36	1.70	−0.28	0.006

The destination-specific contrast was estimated using an unadjusted OLS difference-score model: *b* = 1.481, SE = 0.196, *t*_(375)_ = 7.55, 95% CI [1.095, 1.866], *p* < 0.001.

The fixed-budget design makes two distinguishable facts visible, and they should be read separately. Arm 1 allocated 1.138 more tokens to music education than Arm 2. This difference decomposes exactly into 0.796 additional tokens allocated to the two beneficiaries overall, which came out of what participants retained for themselves, plus 0.343 tokens redirected from poverty relief. In adjusted destination-specific models, the Arm 1 vs. Arm 2 coefficient was 1.110 for music education, 95% CI [0.815, 1.405], *p* < 0.001, and −0.352 for poverty relief, 95% CI [−0.602, −0.103], *p* = 0.006. The result, therefore, reflects both a higher total allocation and a change in its composition. H9a was not supported; H9b was supported descriptively as a fixed-budget composition shift. The analyses do not establish why the destination-specific shift occurred. Figure 5B shows both facts: total allocation rises in Arm 1 while the composition of giving shifts toward the music-education beneficiary. The finding also underscores the value of designating total allocated tokens as the primary outcome and the destination split as the scope test: had allocation to the music-unrelated charity been treated as the sole dependent variable, the study would have recorded a spurious negative effect arising from budget substitution and not from any reduction in prosociality. The allocation geometry in Figure 5A makes this visible; the Arm 1 centroid is displaced toward the music-charity vertex, not toward the self-retention vertex.

### 4.4 Secondary outcomes, robustness, and diagnostics

The secondary unpaid-helping outcome pointed in the same direction but did not differ detectably between conditions. Willingness to complete the task was 51.6% in Arm 1 and 46.0% in Arm 2, OR = 1.20, 95% CI [0.79, 1.81], *p* = 0.39. The interval is wide enough to include both a null and a moderate positive effect, so this outcome neither corroborates nor contradicts the allocation result. Because H1 concerns a hypothetical charitable allocation, this result does not alter its status, but it limits generalization beyond the allocation task.

Two rival explanations were examined. Adding positive and negative state affect and received feedback to the outcome model left the condition coefficient essentially unchanged (*b* = 0.60, *p* = 0.002) and left the social-identity path positive (*b* = 0.61, *p* < 0.001 with the rivals included); the presence-to-allocation path remained non-significant (*p* = 0.17). The focal coefficients were therefore not materially attenuated by measured mood or by the social reward participants accrued during the intervention week. These covariate-adjusted associations do not establish that social identity causally mediated the effect.

Diagnostic checks supported the adequacy of the estimated models (Table 5). For the primary outcome model, residuals were approximately normal (Shapiro–Wilk *W* = 0.99, *p* = 0.17; Jarque–Bera *p* = 0.16), and the Breusch–Pagan test did not indicate heteroskedasticity (*p* = 0.21). HC3 standard errors were nevertheless retained throughout as a conservative robustness choice. Multicollinearity was negligible, with all variance inflation factors ≤ 1.33. No observation appeared strongly influential: 3.2% of cases exceeded the 4/*n* screening threshold, and the maximum Cook's distance was 0.024. The outcome distribution was mildly right-skewed (skew = 0.16) with 5.3% zero-donors and no ceiling responses, well within the tolerance of the linear model.

**Table 5:** Model diagnostics.

Model	*R* ^2^	Residual normality (Shapiro *p*)	Homoskedasticity (Breusch–Pagan *p*)
Primary outcome (Y)	0.062	0.169	0.211
Presence equation	0.133	0.567	0.473
Identity equation	0.188	0.330	0.787
Outcome equation (full)	0.156	0.087	0.456

Primary model: all VIF ≤ 1.33; maximum Cook's distance = 0.024 (3.2% of cases above 4/*n*). HC3 robust standard errors were used throughout.

The three mediation equations were likewise well-behaved. Each was significant overall (all F-tests *p* < 0.001), with residuals that were approximately normal (Shapiro–Wilk *p* = 0.57, 0.33, and 0.09 for the presence, identity, and outcome equations, respectively) and homoskedastic (Breusch–Pagan *p* = 0.47, 0.79, and 0.46). Explained variance was modest but typical for field-experimental mediation models (*R*^2^ = 0.13, 0.19, and 0.16).

The conclusion for H1 did not depend on the distributional treatment of the bounded outcome. The outcome was rescaled to the unit interval by dividing by 10 and a fractional logit was fitted with a logit link and robust standard errors: the condition coefficient was *b* = 0.327, SE = 0.083, 95% CI [0.165, 0.490], *p* < 0.001, and the average marginal effect was 0.757 tokens, 95% bootstrap CI [0.379, 1.124], which agrees with the linear estimate of 0.758 to three decimal places. A proportional-odds logit gave *b* = 0.677, SE = 0.184, 95% CI [0.316, 1.038], *p* < 0.001, and Mann–Whitney *U* = 21,782, *p* < 0.001, rank-biserial *r* = 0.226, 95% bootstrap CI [0.114, 0.337].

**Figure 6:**
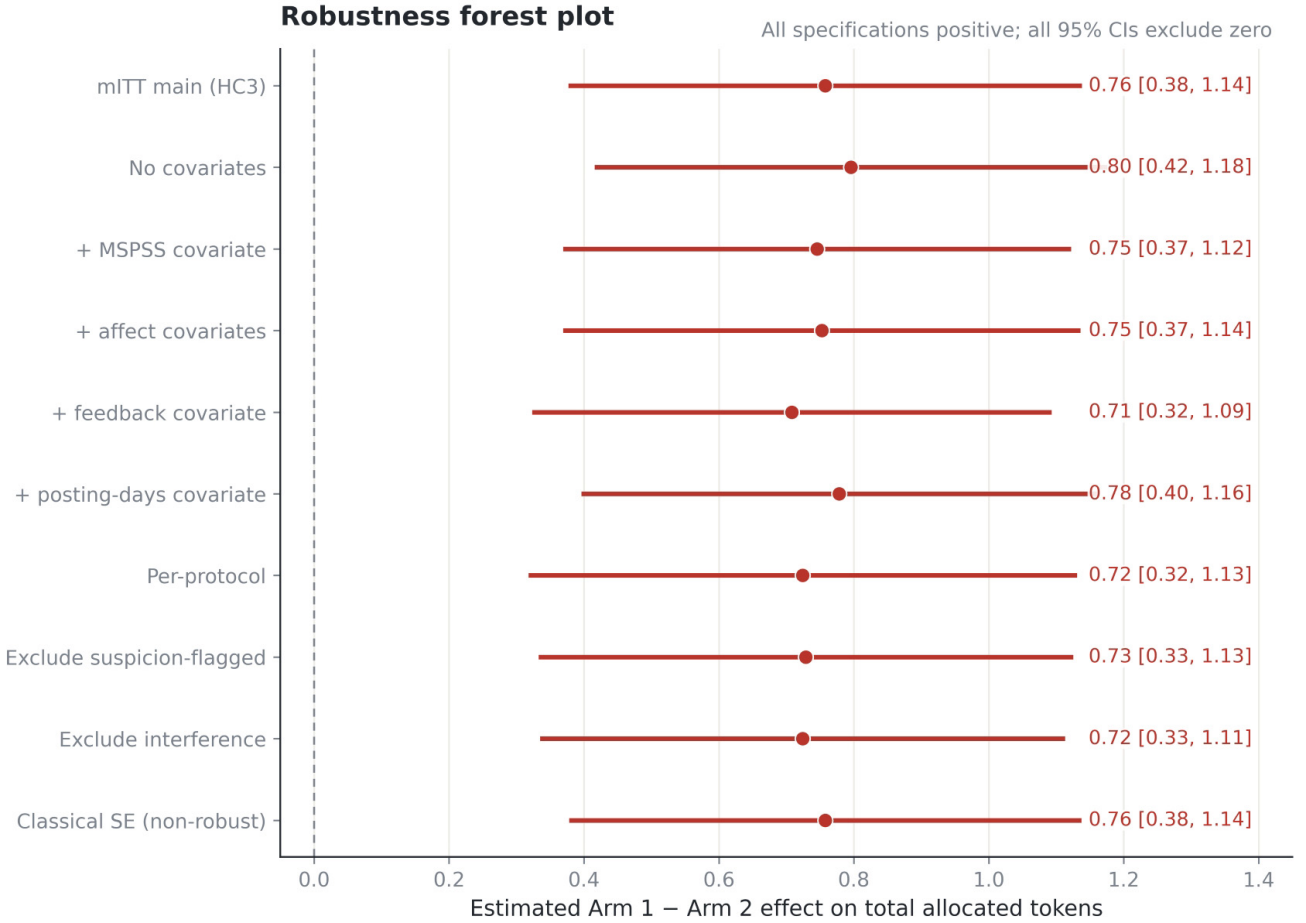
Robustness of the Arm 1 vs. Arm 2 estimate across ten specifications.

In addition to the full intention-to-treat analysis, the focal effect was robust across all ten analytic specifications (Figure 6): the main mITT model, per-protocol, exclusion of suspicion-flagged cases, exclusion of interference-positive cases, addition of affect covariates, addition of feedback covariates, addition of the support covariate, addition of behavioral posting days, and no-covariate and classical (non-robust) variants. Every specification yielded a positive, statistically significant effect of the relational-responsive bundle on allocation, with estimates tightly clustered between 0.70 and 0.80 tokens (median = 0.75). The effect is therefore not an artifact of any single modeling decision. Re-estimation of the full sequential-mediation model on the per-protocol subsample, and after excluding suspicion-flagged and interference-positive cases, left the serial indirect effect and the pattern of moderation unchanged. Table 6 presents the results of the hypothesis tests conducted in this study.

**Table 6:** Hypothesis status.

Row	Result	Status
H1: Bundle effect on hypothetical third-party charitable allocation	*b* =0.758, 95% CI [0.377, 1.138], *d* = 0.425	Supported
Secondary outcome: unpaid helping (not hypothesized)	OR = 1.20, 95% CI [0.79, 1.81], *p* = 0.39	No detectable difference
H2: presence-only association	0.044, 95% CI [−0.035, 0.138]	Not supported
H3: identity-only association	0.028, 95% CI [−0.060, 0.119]	Not supported
H4: serial association	0.053, 95% CI [0.021, 0.099]	Compatible with the proposed serial association
Exploratory H5	a3 = 0.119, 95% CI [−0.032, 0.270]	Not supported
Exploratory H6	a6 = 0.124, 95% CI [−0.038, 0.286]	Not supported
Exploratory H7	c3 = 0.223, 95% CI [−0.192, 0.639]	Not supported
Exploratory H8	All three indices include zero	Not supported
H9a: both beneficiaries	Poverty-relief coefficient = −0.352, 95% CI [−0.602, −0.103]	Not supported
H9b: composition shift	*b* = 1.481, 95% CI [1.095, 1.866]	Supported (descriptive)

Full intention-to-treat robustness analysis. Retaining all 583 randomized participants in their assigned conditions yielded a focal Arm 1 vs. Arm 2 sample of 389. The adjusted allocation effect remained positive, *b* = 0.798, SE = 0.192, *t*_(383)_ = 4.16, *p* < 0.001, 95% CI [0.422, 1.175], *d* = 0.438 (*M* = 4.13 vs. 3.30). In a covariate-adjusted three-arm model retaining all 583 randomized participants in a single specification, the Arm 1 vs. Arm 2 contrast was essentially unchanged, *b* = 0.797, SE = 0.191, 95% CI [0.421, 1.173], *p* < 0.001, and informational posting again did not differ from minimal music use, *b* = −0.066, SE = 0.187, 95% CI [−0.432, 0.301], *p* = 0.725. The serial indirect association remained positive, 0.054, 95% bootstrap CI [0.022, 0.099], whereas the presence-only association, 0.036 [−0.039, 0.121], and identity-only association, 0.020 [−0.070, 0.113], continued to include zero. All three indices of moderated mediation also included zero. The secondary helping result remained non-significant, OR = 1.22, 95% CI [0.81, 1.83], *p* = 0.340. The full ITT analysis, therefore, yielded the same substantive conclusions as the primary mITT analysis (Supplementary Table S30).

## 5 Discussion

### 5.1 Overview of findings

The study asked whether assignment to a relational-responsive music-sharing bundle on social media would increase stated charitable allocation, whether endpoint associations were consistent with the proposed interpersonal pathways, and how any allocation difference was distributed across beneficiaries. The findings support a more limited account than the original moderated dual-pathway model.

Three results anchor this account. First, assignment to the relational-responsive bundle increased total stated allocation relative to informational music posting; informational posting showed no detectable difference from minimal music use. H1 was supported on its specified allocation outcome; the secondary unpaid-helping outcome showed no detectable difference. Second, the specified serial indirect association through perceived social presence and then social identity was positive (H4); the bootstrap intervals for the presence-only and identity-only estimates included zero (H2 and H3). Third, the exploratory analyses detected no moderation by perceived social support (H5-H8). The beneficiary analysis did not support H9a and descriptively supported H9b: under the fixed budget, allocation increased for the music-education beneficiary and decreased slightly for the music-unrelated poverty-relief beneficiary.

The randomized effect was therefore established only for the hypothetical allocation task. The secondary helping outcome did not differ detectably between conditions, and the present evidence does not establish a generalized effect across forms of prosocial behavior.

These findings do not establish that music exposure is ineffective, that any particular relational component produced the focal effect, or that the serial indirect association is an ordered causal mechanism. The randomized contrast identifies the effect of the multicomponent bundle relative to informational music posting. Because both mediators were measured concurrently at the endpoint, their ordering remains theoretical. The beneficiary-composition shift is a separate descriptive result and was not explained by measured social identity. Likewise, the exploratory null moderation results do not show that the associations are independent of participants' support environment.

### 5.2 The primary effect

Random assignment supports a causal effect of assignment to the relational-responsive bundle, relative to informational music posting, on stated charitable allocation in this task. The active arms used the same platform, intervention duration, and daily posting target, but they received different captions and interaction instructions, and behaviorally recorded posting days were not equivalent. Adjustment for recorded posting days left the focal estimate essentially unchanged, but the conditions should not be described as fully matched on dosage.

The estimate for informational music posting vs. minimal music use was close to zero. This secondary contrast does not establish that music exposure is generally ineffective or that relational enactment, and not musical content, caused the focal difference. Informational posting was not a pure exposure condition, and a null contrast cannot identify the active component of the relational-responsive bundle. The result should therefore be interpreted at the level of the assigned bundle.

Prior research has linked prosocial lyrics to helping and donation outcomes (Greitemeyer, 2009a,b; Hong et al., 2023), as well as to outcomes such as tipping and reduced discrimination (Jacob et al., 2010; Greitemeyer and Schwab, 2014). Effects are not uniform: Ruth and Schramm (2021) found effects on prosocial thoughts and empathic emotions, but no significant effect on charitable donation. Music-induced mood has also been related to willingness to help and prosocial decision-making (Fried and Berkowitz, 1979; Wu et al., 2025); synchronous music-making or movement has been linked to cooperation and bonding (Kirschner and Tomasello, 2010; Mogan et al., 2017; Wiltermuth and Heath, 2009). Evolutionary accounts have proposed music as a coevolved system for social bonding (Cross, 2001; Savage et al., 2021).

Much of this literature varies in lyrical content, musical structure, affective qualities, or synchrony. In the present study, both active arms involved music posting; assignment instead varied caption framing and whether responsive interaction was required. The study, therefore, addresses mediated interaction conducted through music. This emphasis is consistent with social-grooming accounts, which treat self-disclosure and responsive attention as relational signals whose functions are not reducible to information transmission alone (Donath, 2007; Tufekci, 2008; Lin, 2019).

Two design features limit the inference. First, the manipulation combined relational framing, emotional self-disclosure, and responsive interaction, so their individual contributions cannot be separated. Second, the study did not include a relational non-musical condition. A similar effect might arise when relational self-disclosure and responsive attention are implemented through text, photographs, or other content, and social-grooming associations have been documented in text- and photo-sharing contexts (Lin, 2019; Lin and Hsieh, 2021; Wang and Wang, 2025). Curzel et al. (2024) found that simulated shared listening increased musical pleasure, but social condition did not directly affect ultimatum offers, dictator-game donation, or helping time, although pleasure predicted Ultimatum offers. This evidence does not demonstrate a direct prosocial effect of unframed shared listening. The present experiment consequently identifies the effect of a relational-responsive interaction bundle implemented through music, not the unique contribution of music.

### 5.3 The mediating pathways

The mediation results did not provide clear support for two independent indirect pathways. The bootstrap intervals for the presence-only and identity-only estimates included zero; the interval for the specified serial product excluded zero. This pattern is compatible with the hypothesized presence-to-identification ordering, but it does not show that the mediators form a single ordered mechanism.

Social presence concerns the sense of being with another in mediated interaction (Biocca et al., 2003; Harms and Biocca, 2004) and has been linked to contribution in online communities (Shen et al., 2010). Self-categorization theory provides a basis for hypothesizing that contextually salient interaction may facilitate identification (Turner, 1985), but it does not logically require social presence to precede identification. The proposed ordering is therefore theoretically plausible; it is not established by the present data.

Prior music-sharing research has treated social presence and identification as antecedents of sharing intentions (Lee et al., 2011; Liu and Luo, 2023). The present findings raise the possibility that repeated presence-laden exchange may also be associated with social identity. Because both mediators were measured concurrently at the endpoint, reverse ordering, reciprocal influence, and shared unmeasured causes remain possible. Temporally separated measurement or mediator manipulation would be needed to test sequence and causal mediation (Bullock et al., 2010; Pirlott and MacKinnon, 2016).

Because the mediators were assessed only once, the study cannot determine whether their magnitudes or ordering would change over a longer intervention. The serial estimate should therefore be treated as an associational endpoint pattern that requires temporal replication.

### 5.4 Perceived social support as a boundary condition

No interaction term or index of moderated mediation excluded zero. Because H5–H8 were exploratory and the focal subsample was not powered to detect small interaction effects, these null results are inconclusive. They provide no evidence that perceived social support moderated the focal pathways, but they do not establish the absence of moderation.

Perceived social support was assessed at baseline as a broad appraisal of available support (Zimet et al., 1988); the proposed mediators were week-specific endpoint perceptions. This conceptual distance may have reduced the moderator's relevance to the manipulated behavior, but the present data do not test that explanation. Need for privacy has been conceptualized as a personality trait (Frener et al., 2024) and was found to moderate the association between live-photo grooming and wellbeing in one cross-sectional study (Wang and Wang, 2025). Privacy- and disclosure-related dispositions are therefore candidates for future research, not established replacements for perceived social support.

The present evidence thus does not support perceived social support as an empirically established boundary condition for this model. Future studies should test theoretically proximal moderators with adequate power and prespecified interaction effects before drawing conclusions about who is most responsive to relational music sharing.

### 5.5 Beneficiary composition and domain-congruent allocation

Assignment to the relational-responsive bundle increased allocation to the music-education beneficiary and slightly reduced allocation to the music-unrelated poverty-relief beneficiary under the fixed budget. We describe this destination-specific pattern as domain-congruent allocation without assigning it a mechanism. H9a was not supported, while H9b was supported descriptively.

An explanation based on measured social identity was anticipated but not supported. The social-identity measure referred to interaction partners encountered during the intervention week; the music-education beneficiary was not that set of people. Social identity was positively associated with total allocation, but direct tests provided no evidence that it explained the beneficiary-composition shift. Domain congruence and identity congruence should therefore not be treated as equivalent.

Pavey et al. (2011) showed that highlighting relatedness increased volunteering intentions and charitable donation, but their studies did not compare allocation across beneficiary domains and therefore do not support a prediction of indiscriminate giving. Hackel et al. (2017) provide evidence that identification can shape target-specific valuation. In the present study, however, the measured identification target and the favored beneficiary were distinct. The result combines higher total allocation with redistribution toward the music-related cause, but the mechanism of that composition shift remains unidentified.

Greater accessibility or salience of the music-related cause could produce the pattern without a group-identification process. Domain salience was not measured, so this explanation remains untested. The fixed budget adds a further constraint: increasing allocation to one destination necessarily limits the resources available for the others. Independent budgets would be needed to distinguish an expansion of concern from a redirection of resources across beneficiaries.

### 5.6 Contributions

The study makes four bounded contributions. First, it brings an asynchronous, user-generated social-media practice into research on music-related prosociality. Much prior work has examined exposure to musical content or co-present and synchronous musical activity (Greitemeyer, 2009a,b; Kirschner and Tomasello, 2010; Mogan et al., 2017; Wiltermuth and Heath, 2009). The present study instead examines music sharing within an existing social network, a form of musical sociality documented in digital music practices (Hagen and Lüders, 2017). The contribution concerns a practice conducted through music, not an isolated effect of music itself.

Second, the study applies a social-grooming perspective to music sharing. Social-grooming accounts frame posting, self-disclosure, and responsive attention as signals that help maintain social ties (Donath, 2007; Tufekci, 2008; Lin, 2019). Music is a plausible vehicle for such relational work because musical taste can signal value similarity (Boer et al., 2011), shared musical activity is associated with perceived relationship commitment (Harwood and Wallace, 2022), and co-experienced music can create a sense of shared feeling (McGuiness and Overy, 2011). The present findings show that assignment to a relational-responsive bundle implemented through music differed from informational posting, while leaving the contribution of the medium itself unresolved.

Third, the study complements research that treats social presence and identification as antecedents of music-sharing intentions (Lee et al., 2011; Liu and Luo, 2023). Here, they were examined as endpoint states within a randomized sharing intervention. The results motivate a testable presence-to-identification hypothesis, while the concurrent measurement prevents that ordering from being claimed as a demonstrated sequence.

Fourth, the analysis distinguishes the level of charitable allocation from its composition. The intervention bundle was associated with both higher total allocation and a shift toward one beneficiary, yet measured social identity did not explain the latter pattern. Treating amount and destination as separate outcomes clarifies that an account of increased giving need not also explain where the additional allocation goes.

### 5.7 Limitations and future research

Several limitations qualify these contributions. The manipulation combined relational framing, emotional self-disclosure, and responsive interaction, so the study cannot isolate their individual effects. No song-level information was retained; therefore, the arms could not be compared on lyrics, genre, valence, arousal, nostalgia, familiarity, or thematic content. Differences in participants' self-selected music are consequently an unmeasured component of the treatment bundle.

The active arms shared the same daily posting target, and self-reported dosage was nearly identical and met the prespecified equivalence criterion, but behaviorally recorded posting days were not equivalent. Adjustment for recorded posting days did not materially change the condition estimate, yet the residual dosage difference remains part of the implemented treatment contrast.

The mediators were measured, not manipulated, and were assessed concurrently at the endpoint. The indirect estimates are therefore associational and cannot identify a causal or temporal mechanism (Bullock et al., 2010; Pirlott and MacKinnon, 2016). The moderation tests were exploratory and not powered for small interactions, so their null results should not be treated as evidence that perceived social support is irrelevant.

The outcome was a simulated, fixed-budget allocation task. It measured stated allocation away from the self, not an implemented charitable transfer, and the shared budget mechanically linked the two beneficiary amounts. The caption indicators were coded independently by two trained coders who were not provided with condition labels; however, the content of the posts may have made the assigned condition apparent. In addition, no formal intercoder-reliability coefficient was computed, and the rater-level records required to reconstruct one were not retained. The coded indicators are therefore interpreted cautiously and only in conjunction with the self-reported manipulation checks. Self-reported manipulation checks also remain vulnerable to common-method and social-desirability bias.

Finally, the sample was young, Chinese, and drawn from one closed social-media platform. Generalizability across platforms and cultures remains uncertain because music-sharing practices and motives may vary by platform and cultural context (Hagen and Lüders, 2017; Liu and Luo, 2023).

Future experiments should decompose the treatment bundle. Within music-sharing conditions, a factorial design crossing caption relationality with required responsive interaction could identify which relational components are active. A separate design crossing content type (musical vs. non-musical) with communication framing (relational vs. informational), while matching dosage and retaining song-level information, could test whether music contributes beyond relational communication through other content. A longer design with temporally separated mediator assessments or direct mediator manipulation would permit a stronger test of the proposed presence-to-identification ordering. Boundary-condition studies should use larger samples and prespecified tests of proximal privacy- or disclosure-related dispositions. Actual or incentive-compatible charitable choices, independent endowments for each beneficiary, and prospective measurement or manipulation of domain salience would help distinguish general increases in giving from beneficiary-specific redirection.

## 6 Conclusion

This randomized field experiment shows that assignment to a 7-day relational-responsive music-sharing bundle, compared with informational music posting, increased stated allocation to two third-party charitable beneficiaries in a simulated fixed-budget task. The result identifies the effect of the multicomponent bundle, not the unique contribution of music, relational framing, self-disclosure, or responsive interaction. Endpoint associations among perceived social presence, social identity, and allocation were consistent with the proposed presence-to-identification ordering, but concurrent measurement does not establish a temporal or mediator-causal sequence. The beneficiary result combined an increase in total allocation with a shift toward the music-education beneficiary; measured social identity did not explain that composition shift. Future factorial and temporally separated designs are needed to isolate active components and test the proposed process.

## Data Availability

The raw data supporting the conclusions of this article will be made available by the authors, without undue reservation.
