# Supplementary material for: Relational-responsive music sharing and third-party charitable allocation: a 7-day randomized field experiment on social media
Source: Front Psychol. 2026 Aug 28;17:1932126. doi: 10.3389/fpsyg.2026.1932126 (PMC13562019; doi:10.3389/fpsyg.2026.1932126)
Supplement: Supplementary file 1 [file Supplementary_file_1.docx]

**Supplementary Material**

All analyses were conducted on the modified intention-to-treat sample (N = 568) unless otherwise stated. Tables are numbered S1–S30. The participant-flow diagram is reported in the main text as Figure 1.

# S1. Participant flow

**Table S1**

*Participant flow from randomization to analysis*

| Stage | Arm 1 | Arm 2 | Arm 3 | Total |
| --- | --- | --- | --- | --- |
| Randomized | 195 | 194 | 194 | 583 |
| Excluded: endpoint attention-check failure | 5 | 7 | 3 | 15 |
| Analysed (mITT) | 190 | 187 | 191 | 568 |
| Focal contrast subsample (Arm 1 vs Arm 2) | 190 | 187 | — | 377 |
| Flagged in suspicion probe (retained) | — | — | — | 33 |
| Flagged for interference (retained) | — | — | — | 19 |
| Per-protocol compliant, focal subsample | 157 | 172 | — | 329 |

Exclusion was not differential across arms, χ²(2) = 1.65, p = .439. The 15 excluded records are endpoint attention-check failures. They are not attrition, dropout, incomplete endpoint surveys, or duplicate responses. No pre-randomization screening counts are reported because the retained records do not support them. All 583 randomized participants were additionally retained in the full intention-to-treat robustness analysis (Section S20).

# S2. Measurement instrument

## S2.1 Translation and contextual adaptation

Two bilingual researchers independently translated and contextually adapted the English-language items into Chinese. They compared their versions against the source wording and agreed on a common administration version.

Adaptation was scale specific. For the social-presence measure, items were adapted from the co-presence and attentional-allocation dimensions of the Networked Minds Social Presence Measure and anchored to the intervention week. For the social-identity measure, the original referent was changed to the people encountered during this week's music-sharing interactions, and this referent was held constant across all four items. Both source measures used seven-point response formats; the present study used five-point formats throughout. No items were added to either scale.

These translation and adaptation procedures are distinct from the derivation of the behavioral coding variables, and they do not supply a content-coding reliability statistic; see Section S19.

## S2.2 Baseline music sharing

Baseline descriptive control. Scale 1 = 从不 (never) to 5 = 总是 (always). Composite: BMS_mean.

**Table S2**

*Baseline music-sharing items*

| **Code** | **中文题项** | **English** |
| --- | --- | --- |
| BMS1 | 过去一个月，我在社交媒体上分享过歌曲、歌单、歌词或与音乐相关的内容。 | In the past month, I shared songs, playlists, lyrics, or music-related content on social media. |
| BMS2 | 过去一个月，我通过点赞、评论或回复回应过他人与音乐相关的动态。 | In the past month, I responded to others' music-related posts through likes, comments, or replies. |
| BMS3 | 过去一个月，我通过分享音乐来表达自己的感受或个人经历。 | In the past month, I used music sharing to express my feelings or personal experiences. |
| BMS4 | 过去一个月，我看到过认识的人分享与音乐相关的内容。 | In the past month, I saw music-related content shared by people I know. |

## S2.3 Perceived social support

MSPSS (Zimet et al., 1988). Twelve items, baseline, original seven-point scale (1 = 极不同意 to 7 = 极同意). Moderator W. Composites: MSPSS_mean; friends subscale MSPSS_friends_mean (SS6, SS7, SS9, SS12).

**Table S3**

*Perceived social support items*

| **Code** | **中文题项** | **English** |
| --- | --- | --- |
| SS1 | 在我需要时，有一个特别的人会在我身边。 | There is a special person who is around when I am in need. |
| SS2 | 有一个特别的人，我可以与他/她分享我的快乐与忧伤。 | There is a special person with whom I can share my joys and sorrows. |
| SS3 | 我的家人真心地想帮助我。 | My family really tries to help me. |
| SS4 | 我从家人那里得到了我所需要的情感帮助与支持。 | I get the emotional help and support I need from my family. |
| SS5 | 有一个特别的人，是我真正的安慰来源。 | I have a special person who is a real source of comfort to me. |
| SS6 | 我的朋友们真心地想帮助我。 | My friends really try to help me. |
| SS7 | 当事情出错时，我可以依靠我的朋友。 | I can count on my friends when things go wrong. |
| SS8 | 我可以和家人谈论我的困扰。 | I can talk about my problems with my family. |
| SS9 | 我有一些朋友，我可以与他们分享我的快乐与忧伤。 | I have friends with whom I can share my joys and sorrows. |
| SS10 | 有一个特别的人，关心我的感受。 | There is a special person in my life who cares about my feelings. |
| SS11 | 我的家人愿意帮助我做决定。 | My family is willing to help me make decisions. |
| SS12 | 我可以和朋友谈论我的困扰。 | I can talk about my problems with my friends. |

## S2.4 Trait prosociality

Prosocialness Scale for Adults (Caprara et al., 2005). Sixteen items, baseline, 1 = 完全不符合 to 5 = 完全符合. Covariate. Composite: Trait_prosociality_mean.

**Table S4**

*Trait prosociality items*

| **Code** | **中文题项** | **English** |
| --- | --- | --- |
| TP1 | 我乐于在朋友或同事的活动中帮助他们。 | I am pleased to help my friends and colleagues in their activities. |
| TP2 | 我会与朋友分享我拥有的东西。 | I share the things that I have with my friends. |
| TP3 | 我努力去帮助别人。 | I try to help others. |
| TP4 | 我愿意参加志愿活动去帮助有需要的人。 | I am available for volunteer activities to help people in need. |
| TP5 | 我对处于困境中的人感同身受。 | I feel empathic toward those who are in need. |
| TP6 | 我会立刻帮助那些有需要的人。 | I help immediately those who are in need. |
| TP7 | 我尽我所能帮助别人避免麻烦。 | I do what I can to help others avoid getting into trouble. |
| TP8 | 我能强烈地感受到别人的感受。 | I intensely feel what others feel. |
| TP9 | 我愿意把自己的知识和能力提供给别人。 | I am willing to make my knowledge and abilities available to others. |
| TP10 | 我会试着安慰那些难过的人。 | I try to console those who are sad. |
| TP11 | 我很容易借钱或借东西给别人。 | I easily lend money or other things. |
| TP12 | 我很容易设身处地为不适的人着想。 | I easily put myself in the shoes of those who are in discomfort. |
| TP13 | 我会试着亲近并照顾那些有需要的人。 | I try to be close to and take care of those who are in need. |
| TP14 | 我很容易与朋友分享好机会。 | I easily share with friends any good opportunity that comes to me. |
| TP15 | 我会花时间陪伴那些感到孤单的朋友。 | I spend time with those friends who feel lonely. |
| TP16 | 即使朋友没有直接说出来，我也能立即察觉他们的不适。 | I immediately sense my friends' discomfort even when it is not directly communicated to me. |

## S2.5 Manipulation check: posting dosage

Endpoint, 1 = 完全不同意 to 5 = 完全同意. Expected pattern Arm 1 ≈ Arm 2 > Arm 3. Composite: MC_posting_mean.

**Table S5**

*Posting-dosage manipulation-check items*

| **Code** | **中文题项** | **English** |
| --- | --- | --- |
| MCB1 | 过去一周，我在社交媒体上分享或转发过与音乐相关的内容。 | During the past week, I shared or reposted music-related content on social media. |
| MCB2 | 过去一周，我在社交媒体上发布过与音乐相关的动态。 | During the past week, I posted music-related updates on social media. |
| MCB3 | 总体而言，过去一周我发布音乐相关内容比平时更多。 | Overall, during the past week I posted about music more than usual. |

## S2.6 Manipulation check: relational-responsive grooming

Endpoint, 1 = 完全不同意 to 5 = 完全同意. Expected pattern Arm 1 > Arm 2 ≈ Arm 3. Composite: MC_relational_mean.

**Table S6**

*Relational-responsive manipulation-check items*

| **Code** | **中文题项** | **English** |
| --- | --- | --- |
| MCR1 | 我发布的音乐内容表达了我的感受，或有助于维系我的人际关系。 | My music posts expressed my feelings or helped maintain my relationships. |
| MCR2 | 我的音乐分享是针对特定的人或特定关系的。 | My music sharing was directed at particular people or particular relationships. |
| MCR3 | 围绕音乐内容，我与他人有来有回地互动过。 | I engaged in back-and-forth interaction with others around music content. |
| MCR4 | 我用具体的评论或回复回应过他人的音乐动态。 | I responded to others' music posts with substantive comments or replies. |

*Note.* MCR3 and MCR4 are components of the intervention bundle rather than confounds. Responsive attention-giving is constitutive of the treatment as defined.

## S2.7 Perceived social presence (M1)

Five items adapted from the co-presence and attentional-allocation dimensions of the Networked Minds measure (Harms and Biocca, 2004). Endpoint, 1 = 完全不同意 to 5 = 完全同意. Referent: 本周的音乐分享互动. Composite: Social_presence_mean.

**Table S7**

*Perceived social presence items*

| **Code** | **中文题项** | **English** |
| --- | --- | --- |
| SP1 | 在本周的音乐分享互动中，我感觉与我互动的人真的“在场”。 | During this week's music-sharing interactions, I felt that the people I interacted with were really “there” with me. |
| SP2 | 与我互动的人的存在感，对我来说是明显的。 | The presence of the people I interacted with was obvious to me. |
| SP3 | 我感觉他们注意到了我。 | I felt that they noticed me. |
| SP4 | 我密切关注着与我互动的人。 | I paid close attention to the people I interacted with. |
| SP5 | 我感觉他们在关注我。 | I felt that they were paying attention to me. |

Note. The original Networked Minds measure used a seven-point response format. The present study used a five-point format and a week-specific interaction referent. This five-item set is neither the complete Networked Minds inventory nor a previously validated five-item short form.

## S2.8 Social identity (M2)

Four items adapted from Lee et al. (2011). Endpoint, 1 = 完全不同意 to 5 = 完全同意. Referent held constant across items: 本周音乐分享互动中接触到的人. Composite: Social_identity_mean.

**Table S8**

*Social identity items*

| **Code** | **中文题项** | **English** |
| --- | --- | --- |
| SI1 | 对于本周音乐分享互动中接触到的人，我有一种归属感。 | I feel a sense of belonging with the people I came into contact with in this week's music-sharing interactions. |
| SI2 | 我对他们有一种在一起或亲近的感觉。 | I feel togetherness or closeness with them. |
| SI3 | 我对他们有强烈的积极情感。 | I have a strong positive feeling toward them. |
| SI4 | 我享受与他们相处。 | I enjoy being together with them. |

*Note.* The four item stems correspond to Lee et al. (2011), but the group referent and response format were adapted for the present study. The referent was held constant across all four items.

## S2.9 State affect

I-PANAS-SF (Thompson, 2007). Endpoint, after the mediators and before the allocation task. Instruction: 请指出你此刻在多大程度上有以下感受. 1 = 几乎没有 to 5 = 非常强烈.

**Table S9**

*State affect items*

| **Code** | **中文** | **English** | **Composite** |
| --- | --- | --- | --- |
| PA1 | 有活力的 | Active | Positive_affect_mean |
| PA2 | 有决心的 | Determined |  |
| PA3 | 专注的 | Attentive |  |
| PA4 | 备受鼓舞的 | Inspired |  |
| PA5 | 警觉的 | Alert |  |
| NA1 | 心烦的 | Upset | Negative_affect_mean |
| NA2 | 有敌意的 | Hostile |  |
| NA3 | 羞愧的 | Ashamed |  |
| NA4 | 紧张的 | Nervous |  |
| NA5 | 害怕的 | Afraid |  |

## S2.10 Feedback received

Endpoint. Used in robustness analyses only; feedback is endogenous to condition and is not treated as a mediator.

**Table S10**

*Feedback items*

| **Code** | **中文题项** | **English** | **Response options** |
| --- | --- | --- | --- |
| FB1 | 过去一周，你的音乐相关动态大约收到了多少个赞？ | Approximately how many likes did your music-related posts receive during the past week? | 0 / 1–5 / 6–15 / 16–30 / 30+ |
| FB2 | 过去一周，你的音乐相关动态大约收到了多少条评论？ | Approximately how many comments did your music-related posts receive during the past week? | 0 / 1–5 / 6–15 / 16–30 / 30+ |
| FB3 | 我收到的回应是积极的、支持性的。 | The feedback I received was positive and supportive. | 1–5 |

## S2.11 Primary outcome: the allocation task

Presented as a separate module after a study-complete transition page. The allocation rule was shown before the decision screen.

中文指导语：感谢你完成本研究。请设想你获得了 10 枚代币，每枚代币的名义价值为人民币 1 元。你可以自行决定如何分配这 10 枚代币：留给自己、捐给「乡村音乐教育公益项目」、或捐给「综合扶贫慈善项目」。三项之和必须等于 10。

English: Thank you for completing the study. Imagine that you have received 10 tokens, each with a nominal value of 1 RMB. You may decide how to divide these 10 tokens between yourself, a rural music-education public-interest programme, and a general poverty-relief charity. The three amounts must sum to 10.

**Table S11**

*Allocation-task fields*

| **Field** | **中文** | **English** | **Variable** |
| --- | --- | --- | --- |
| 1 | 留给自己 | Keep for myself | tokens_kept_self |
| 2 | 捐给「乡村音乐教育公益项目」 | Allocate to the rural music-education programme | tokens_donated_music_education |
| 3 | 捐给「综合扶贫慈善项目」 | Allocate to the general poverty-relief charity | tokens_donated_neutral_charity |

*Note.* Primary outcome tokens_donated_total_primary_Y = field 2 + field 3, under the constraint field 1 + field 2 + field 3 = 10. The task was simulated: the tokens carried no implemented monetary conversion, were not added to participant compensation, and generated no transfer to either beneficiary.

## S2.12 Secondary outcome: unpaid helping request

中文：我们正在为另一项研究评估一些简短材料。这项任务完全自愿，与你的报酬无关，大约需要 3 分钟。你是否愿意参与？

English: We are evaluating some short materials for another study. This task is entirely voluntary and unrelated to your payment, and takes about 3 minutes. Would you be willing to take part?

Recorded as helping_request_yes (binary) and helping_materials_completed (count).

## S2.13 Intervention instruction cards and daily log

**Table S12**

*Intervention instruction cards by arm*

| **Condition** | **中文指导语** | **English** |
| --- | --- | --- |
| Arm 1 Relational-responsive music sharing | 请每天至少在朋友圈发布 1 条与音乐相关的内容（歌曲、歌单、歌词、音乐视频或与音乐有关的回忆），并配上个人化、情感性或关系性的文字，把这段音乐与某段回忆、某种情绪、某个人、某段关系或某次共同经历联系起来。此外，请每天至少用具体的评论或回复回应他人的一条音乐相关动态（仅点赞不算）。 | Post at least one music-related item on Moments each day for seven days. Give each post a personal, emotional, or relational caption connecting the music to a memory, an emotion, a person, a relationship, or a shared experience. Respond each day to at least one music-related post by someone else with a substantive comment or reply; a bare like does not count. |
| Arm 2 Informational music posting | 请每天至少在朋友圈发布 1 条与音乐相关的内容，并配上信息性的文字，例如歌曲事实、流派、艺人、发行年份、榜单表现、编曲或节奏等客观描述。请不要加入个人回忆、情感自我表露、献给某人的话语，或与人际关系有关的内容。 | Post at least one music-related item each day for seven days with an informational caption — artist, genre, release year, arrangement, rhythm, or chart performance. Avoid personal memories, emotional self-disclosure, dedications, and relationship-oriented framing. |
| Arm 3 Minimal music use | 请像平常一样使用朋友圈，浏览和互动都保持日常习惯，但请不要发布任何与音乐相关的内容。 | Use Moments as you normally would, browsing and interacting as usual, but do not post any music-related content. |

**Table S13**

*Daily micro-log items*

| **#** | **中文** | **English** |
| --- | --- | --- |
| 1 | 今天你是否发布了与音乐相关的内容？ | Did you post music-related content today? |
| 2 | 你分享的是什么？ | What did you share? |
| 3 | 请粘贴你使用的完整文案。 | Please paste the exact caption you used. |
| 4 | 今天你是否回应了他人的音乐相关动态？如果有，是以何种方式？ | Did you respond to others' music-related posts today, and if so how? |
| 5 | 完成今天的任务有困难吗？ | Did you have any difficulty completing today's task? |

*Note.* Item 3 recorded caption text only. The retained analytic file holds participant-level aggregate codes derived from these logs; the original logs and privacy-screened screenshots were deleted after coding under the study's privacy protocol.

## S2.14 Attention checks

**Table S14**

*Attention checks*

| **Point** | **中文** | **English** | **Variable** |
| --- | --- | --- | --- |
| Baseline | 为确认你在认真作答，请在此题选择「不同意」。 | To confirm you are reading carefully, please select 'disagree' for this item. | screening |
| Endpoint | 请在此题选择「有些同意」。 | Please select 'somewhat agree' for this item. | attention_t1_pass |

*Note.* Fifteen participants failed the endpoint check and were excluded from the primary mITT analysis; they were retained in the full intention-to-treat robustness analysis (Section S20).

# S3. Reliability

**Table S15**

*Cronbach's alpha with bootstrap confidence intervals*

| **Construct** | **Items** | **α** | **95% CI** |
| --- | --- | --- | --- |
| Perceived social presence | 5 | .788 | [.758, .815] |
| Social identity | 4 | .774 | [.741, .799] |
| Perceived social support | 12 | .920 | [.910, .929] |
| Trait prosociality | 16 | .903 | [.890, .914] |
| Positive affect | 5 | .706 | [.671, .738] |
| Negative affect | 5 | .795 | [.764, .821] |

*Note.* Composite reliability is reported separately in Table S18 from the confirmatory factor model.

# S4. Confirmatory factor analysis

Models were estimated by maximum likelihood on all 47 indicators (N = 568).

**Table S16**

*Confirmatory factor analysis, hypothesized and alternative models*

| **Model** | **χ²** | **df** | **χ²/df** | **CFI** | **TLI** | **RMSEA** |
| --- | --- | --- | --- | --- | --- | --- |
| Six-factor (hypothesized) | 1141.89 | 1019 | 1.12 | .986 | .985 | .015 |
| One-factor (all 47 indicators) | 6034.92 | 1034 | 5.84 | .426 | .400 | .092 |

*Note.* The one-factor model is the formal test of the possibility that a set of positively valenced social constructs collapses into a single factor. It does not fit; the six-factor structure is retained.

**Table S17**

*Discriminant validity of the two focal mediators*

| **Model (9 presence and identity items)** | **χ²** | **df** | **p** | **CFI** | **TLI** | **RMSEA** |
| --- | --- | --- | --- | --- | --- | --- |
| Two-factor: presence and identity distinct | 35.41 | 26 | .103 | .993 | .990 | .025 |
| One-factor: presence and identity combined | 353.19 | 27 | < .001 | .759 | .679 | .146 |

*Note.* Nested comparison Δχ²(1) = 317.78, p < .001.

**Table S18**

*Standardized loadings, composite reliability, and average variance extracted*

| **Construct** | **Loading range** | **CR** | **AVE** |
| --- | --- | --- | --- |
| Perceived social presence | .641–.674 | .789 | .427 |
| Social identity | .655–.699 | .774 | .461 |
| Perceived social support | .668–.734 | .921 | .491 |
| Trait prosociality | .561–.664 | .903 | .369 |
| Positive affect | .553–.587 | .707 | .325 |
| Negative affect | .627–.690 | .795 | .437 |

# S5. Discriminant validity

**Table S19**

*Heterotrait–monotrait ratios*

| **Construct pair** | **HTMT** |
| --- | --- |
| Presence–identity | .505 |
| Presence–support | .29 |
| Identity–support | .30 |
| Presence–trait prosociality | .19 |
| Identity–trait prosociality | .19 |
| Support–trait prosociality | .22 |
| Presence–positive affect | .22 |
| Identity–positive affect | .19 |
| All pairs involving negative affect | ≤ .12 |

*Note.* The largest ratio is between the two focal mediators at .505, well below the conservative .85 criterion. The attenuation-corrected latent correlation between presence and identity is .506.

**Table S20**

*Fornell–Larcker discriminant-validity comparison, confirmatory basis*

| **Construct** | **1** | **2** | **3** | **4** | **5** | **6** |
| --- | --- | --- | --- | --- | --- | --- |
| 1. Perceived social presence | **.654** |  |  |  |  |  |
| 2. Social identity | .395 | **.679** |  |  |  |  |
| 3. Perceived social support | .250 | .247 | **.701** |  |  |  |
| 4. Trait prosociality | .155 | .160 | .206 | **.607** |  |  |
| 5. Positive affect | .174 | .149 | .004 | .000 | **.570** |  |
| 6. Negative affect | −.093 | −.070 | −.000 | −.090 | −.053 | .661 |

***Note.*** Diagonal entries (bold) are square roots of average variance extracted from the six-factor confirmatory model (Table S18); off-diagonal entries are correlations among construct composites. The criterion is satisfied in every row: each diagonal entry exceeds the absolute value of every correlation involving that construct. The largest correlation in the matrix is .395, between the two focal mediators.

# S6. Common method variance

The first unrotated component accounts for 17.9% of total variance. This diagnostic is reported for completeness and does not rule out common-method bias; Harman's single-factor test has low sensitivity and its result should not be presented as evidence that method variance is absent. The stronger evidence is the one-factor confirmatory model in Table S16, which fits poorly, and the discriminant results in Table S19.

# S7. Multicollinearity

**Table S21**

*Variance inflation factors, primary outcome model*

| **Predictor** | **VIF** |
| --- | --- |
| Condition | 1.33 |
| Trait prosociality | 1.02 |
| Baseline music-sharing frequency | 1.03 |
| Age | 1.01 |
| Gender | 1.33 |

# S8. Dosage equivalence by indicator

Equivalence was tested by two one-sided tests against a prespecified smallest effect size of interest of d = ±0.30.

**Table S22**

*Equivalence tests for posting dosage, Arm 1 versus Arm 2*

| **Indicator** | **M₁** | **M₂** | **Diff.** | **d** | **90% CI** | **p(TOST)** | **Equivalence** |
| --- | --- | --- | --- | --- | --- | --- | --- |
| Self-reported posting dosage (1–5) | 3.725 | 3.731 | −0.006 | −0.011 | [−0.102, 0.089] | .003 | Established |
| Behavioral posting days (0–7) | 6.211 | 5.936 | +0.275 | +0.314 | [0.126, 0.423] | .554 | Not established |
| Behavioral days, bounds widened to ±0.40 | 6.211 | 5.936 | +0.275 | +0.314 | [0.126, 0.423] | .202 | Not established |

*Note.* The behavioral difference is also significantly non-zero, t(375) = 3.05, p = .002. Equivalence is claimed only for the self-report indicator.

**Table S23**

*Whether the residual dosage difference accounts for the treatment estimate*

| **Model** | **b (condition)** | **SE** | **95% CI** | **p** |
| --- | --- | --- | --- | --- |
| Primary model | 0.758 | 0.193 | [0.377, 1.138] | < .001 |
| Adding behavioral posting days | 0.778 | 0.194 | [0.396, 1.160] | < .001 |

*Note.* Posting days does not predict allocation with condition in the model, b = −0.076, 95% CI [−0.286, 0.133], p = .474.

# S9. Secondary contrast: Arm 2 versus Arm 3

**Table S24**

*Informational posting versus minimal use, total tokens allocated*

| Statistic | Value |
| --- | --- |
| n | 378 |
| M Arm 2 / M Arm 3 | 3.283 / 3.393 |
| b (adjusted) | −0.082, SE 0.190 |
| 95% CI | [−0.455, 0.292] |
| t / p | −0.43 / .668 |
| Cohen's d | −0.059 |
| Post hoc equivalence against d = ±0.30 | p = .010 |

*Note.* The equivalence test on this contrast is post hoc. The ±0.30 bound was prespecified for the dosage manipulation check, and there is no record that it was fixed for this outcome comparison before that comparison was examined. It is reported as a sensitivity analysis.

# S10. Bounded-outcome and distribution-free models

**Table S25**

*The treatment estimate under four models*

| **Model** | **Estimate** | **SE** | **95% CI** | **p** | **Scale** |
| --- | --- | --- | --- | --- | --- |
| OLS, HC3 (primary) | 0.758 | 0.193 | [0.377, 1.138] | < .001 | tokens |
| Fractional logit, coefficient | 0.327 | 0.083 | [0.165, 0.490] | < .001 | logit |
| Fractional logit, average marginal effect | 0.757 | — | [0.379, 1.124] | < .001 | tokens |
| Proportional-odds logit | 0.677 | 0.184 | [0.316, 1.038] | < .001 | logit |
| Mann–Whitney, rank-biserial r | .226 | — | [.114, .337] | < .001 | rank |

# S11. Mediator ordering

Both mediators were measured in the same endpoint survey and neither was randomized. The design cannot establish temporal precedence.

**Table S26**

*Serially ordered indirect associations under both orderings*

| **Specification** | **Indirect association** | **95% bootstrap CI** |
| --- | --- | --- |
| Presence → identity (proposed ordering) | 0.0535 | [0.0201, 0.0990] |
| Identity → presence (reverse) | 0.0061 | [−0.0049, 0.0229] |

*Note.* Both mediators were measured concurrently at endpoint. The comparison is a sensitivity analysis of alternative statistical orderings and does not establish temporal precedence.

# S12. Beneficiary composition

Because the two beneficiary amounts come from the same fixed 10-token budget, the destination-specific contrast is estimated by regressing the within-participant difference (music-education minus poverty-relief tokens) on condition. This is algebraically equivalent to the condition-by-beneficiary interaction for two repeated destinations, and it avoids a random-intercept specification that is not identified for a fixed-sum outcome: under a fixed budget the two amounts are negatively correlated within person, which a non-negative random-intercept variance cannot represent.

**Table S27**

*Effects on every component of the fixed budget*

| **Outcome** | **M Arm 1** | **M Arm 2** | **Raw diff.** | **b (adj.)** | **95% CI** | **p** |
| --- | --- | --- | --- | --- | --- | --- |
| Tokens kept by the participant | 5.921 | 6.717 | −0.796 | −0.758 | [−1.138, −0.377] | < .001 |
| Total allocated to beneficiaries | 4.079 | 3.283 | +0.796 | +0.758 | [0.377, 1.138] | < .001 |
| Music-education programme | 2.721 | 1.583 | +1.138 | +1.110 | [0.815, 1.405] | < .001 |
| Poverty-relief charity | 1.358 | 1.701 | −0.343 | −0.352 | [−0.602, −0.103] | .006 |

*Note.* The decomposition is exact. The music-education programme gained 1.138 tokens: 0.796 from tokens the participant would otherwise have kept, and 0.343 redirected from the poverty-relief beneficiary. Redistribution between beneficiaries cannot reduce the retained share.

**Table S28**

*The destination-specific contrast*

| **Specification** | **b** | **SE** | **t** | **df** | **95% CI** | **p** |
| --- | --- | --- | --- | --- | --- | --- |
| Difference score, unadjusted | 1.481 | 0.196 | 7.55 | 375 | [1.095, 1.866] | < .001 |
| Difference score, covariate-adjusted | 1.462 | 0.199 | 7.34 | 371 | [1.071, 1.853] | < .001 |

*Note.* Mean difference score: +1.363 in Arm 1, −0.118 in Arm 2.

# S13. Does the beneficiary shift track measured social identity?

**Table S29**

*Tests of the identity account of beneficiary composition*

| **Test** | **Estimate** | **SE** | **95% CI** | **p** |
| --- | --- | --- | --- | --- |
| Condition × identity on (music − poverty relief) | 0.359 | 0.259 | [−0.151, 0.868] | .167 |
| Condition × identity on the music-education share | 0.017 | 0.044 | [−0.069, 0.103] | .692 |
| Main effect of identity on (music − poverty relief) | 0.011 | 0.169 | [−0.322, 0.344] | .948 |
| Indirect: condition → identity → difference score | 0.027 | — | [−0.010, 0.087] | ns |
| Serial: condition → presence → identity → difference | 0.025 | — | [−0.004, 0.062] | ns |

*Note.* None of these tests supports the identity account. The condition effect on the difference score is essentially undiminished by identity, b = 1.436, 95% CI [1.040, 1.832]. Social identity remains positively associated with total allocation within the serial model (b = 0.582, p < .001) but does not track the destination-specific shift.

# S14. Unmeasured properties of the shared music

The retained analytic dataset contains no song-level information: no title, link, lyric, exact caption, artist, genre, valence, arousal, nostalgia, familiarity, or thematic variable. The privacy-screened screenshots and original micro-logs were deleted after the planned aggregate coding. A valid post hoc comparison of the musical materials across arms is therefore not possible, and condition-related differences in the music selected remain a rival explanation.

Coefficient-stability diagnostics such as Oster’s δ and the E-value address selection on unobservables in observational designs rather than unmeasured treatment composition in a randomized experiment; they therefore do not address this limitation.

# S15. Per-protocol analysis

Arm 1: posted on ≥ 5 of 7 days, captions coded relational on ≥ 50% of posting days, and responded on ≥ 4 of 7 days. Arm 2: posted on ≥ 5 of 7 days and mean coded informationality ≥ 1.0 on the 0–2 scale.

# S16. Robustness across specifications

All ten estimates are positive and all ten 95% confidence intervals exclude zero, with point estimates between 0.708 and 0.796. The specifications are: mITT main (HC3); per-protocol; excluding suspicion-flagged cases; excluding interference-positive cases; adding state affect covariates; adding the feedback covariate; adding perceived social support; adding behavioral posting days; no covariates; and classical standard errors. An additional outlier-exclusion specification (residuals beyond three standard deviations) removed no cases and is identical to the main model; it is therefore not displayed as a separate specification. A full intention-to-treat robustness analysis is reported separately in Section S20. See main-text Figure 6.

# S17. Statistical power

Sample-size planning targeted the primary Arm 1 versus Arm 2 contrast. The focal subsample of n = 377 affords 80% power at α = .05, two-tailed, to detect d ≥ 0.289; the observed effect is d = 0.425. Planning did not establish power for interactions or moderated indirect associations, which for comparable standardized magnitudes require roughly four times the sample size of a main effect. Perceived social support was therefore examined as an exploratory boundary condition throughout, with confidence intervals reported for every term.

# S18. Model diagnostics

Primary outcome model: R² = .062, Shapiro–Wilk p = .169, Breusch–Pagan p = .211, maximum Cook's distance 0.024, residual skew 0.133. Presence equation R² = .133; identity equation R² = .188; outcome equation R² = .156. The outcome distribution is mildly right-skewed with 5.3% zero allocations and no ceiling responses. HC3 standard errors are used throughout.

# S19. Coding of caption variables and reliability reporting

Captions recorded in the daily logs and verified against privacy-screened screenshots were coded for relationality, emotionality, informationality, and responsiveness by two trained coders who were not provided with participants’ randomized condition labels. Because condition-specific instructions could nevertheless be inferred from post content, the procedure is not treated as successful masking of condition assignment. The coders worked independently and resolved disagreements through discussion before codes were aggregated to the participant level. A formal intercoder-reliability coefficient was not computed and cannot be reconstructed because rater-level records were not retained. The coded indicators are therefore treated as complementary behavioral evidence and interpreted jointly with the self-reported manipulation checks.

The coded variables converge with the independently measured self-report manipulation check: coded relationality separated the two active arms at d = 2.91 and the self-reported relational-responsive index at d = 1.69, in the same direction.

# S20. Full intention-to-treat robustness analysis

**Table S30**

*Full intention-to-treat robustness analysis*

| **Result** | **Primary mITT** | **Full ITT** |
| --- | --- | --- |
| Randomized participants | 583 | 583 |
| Participants included in analysis, all three arms | 568 | 583 |
| Focal Arm 1 versus Arm 2 analysis, n | 377 | 389 |
| Mean allocation, Arm 1 / Arm 2 | 4.08 / 3.28 | 4.13 / 3.30 |
| Adjusted condition effect, b (SE) | .758 (.193) | .798 (.192) |
| 95% CI | [.377, 1.138] | [.422, 1.175] |
| Cohen’s d | 0.425 | 0.438 |
| Secondary contrast (Arm 2 vs Arm 3), b | −.08 [−.46, .29], p = .668 | −.066 [−.432, .301], p = .725 |
| Presence-only indirect association | .044 [−.035, .138] | .036 [−.039, .121] |
| Identity-only indirect association | .028 [−.060, .119] | .020 [−.070, .113] |
| Serial indirect association | .053 [.021, .099] | .054 [.022, .099] |
| Index via presence | .019 [−.017, .077] | .016 [−.020, .071] |
| Index via identity | .072 [−.014, .177] | .076 [−.011, .182] |
| Serial index | .024 [−.005, .060] | .025 [−.005, .061] |
| Helping outcome | OR = 1.20 [.79, 1.81], p = .39 | OR = 1.22 [.81, 1.83], p = .340 |

*Note.* The full ITT analysis retains all randomized participants in their assigned conditions, including endpoint attention-check failures. Indirect associations and indices are reported with 95% bootstrap confidence intervals.
